# Supplementary material for: Cultural Identity Conflict Informs Engagement with Self-Management Behaviours for South Asian Patients Living with Type-2 Diabetes: A Critical Interpretative Synthesis of Qualitative Research Studies
Source: Int J Environ Res Public Health. 2021 Mar 5;18(5):2641. doi: 10.3390/ijerph18052641 (PMC7967381; doi:10.3390/ijerph18052641)
Supplement: Supplementary file 1 [file ijerph-18-02641-s001.pdf]

Supplementary files

Table S1: The ENTREQ Checklist

**Item**	**Guide and description**	**Reported on page #**
Aim	State the research question the synthesis addresses	3
Synthesis methodology	Identify the synthesis methodology or theoretical framework which underpins the synthesis, and describe the rationale for choice of methodology (e.g. meta- ethnography, thematic synthesis, critical interpretive synthesis, grounded theory synthesis, realist synthesis, meta-aggregation, meta-study, framework synthesis).	3
Approach to searching	Indicate whether the search was pre-planned (comprehensive search strategies to seek all available studies) or iterative (to seek all available concepts until theoretical saturation is achieved).	3-4
Inclusion criteria	Specify the inclusion/exclusion criteria (e.g. in terms of population, language, year limits, type of publication, study type).	4
Data sources	Describe the information sources used (e.g. electronic databases (MEDLINE, EMBASE, CINAHL, psychINFO, Econlit), grey literature databases (digital thesis, policy reports), relevant organisational websites, experts, information specialists, generic web searches (Google Scholar), hand searching, reference lists) and when the searches were conducted; provide the rationale for using the data sources.	4
Electronic Search strategy	Describe the literature search (e.g. provide electronic search strategies with population terms, clinical or health topic terms, experiential or social phenomena related terms, filters for qualitative research and search limits).	3-4 Supplementary file 2
Study screening methods	Describe the process of study screening and sifting (e.g. title, abstract and full text review, number of independent reviewers who screened studies)	4
Study characteristics	Present the characteristics of the included studies (e.g. year of publication, country, population, number of participants, data collection, methodology, analysis, research questions).	5 & 8-12 (Table 1)
Study selection results	Identify the number of studies screened and provide reasons for study exclusion (e.g. for comprehensive searching, provide numbers of studies screened and reasons for exclusion indicated in a figure/flowchart; for iterative searching describe reasons for study exclusion and inclusion based on modifications t the research question and/or contribution to theory development).	6 & Figure 1.

Rationale for appraisal	Describe the rationale and approach used to appraise the included studies or selected findings (e.g. assessment of conduct (validity and robustness), assessment of reporting (transparency), assessment of content and utility of the findings).	4
Appraisal items	State the tools, frameworks and criteria used to appraise the studies or selected findings (e.g. Existing tools: CASP, QARI, COREQ, Mays and Pope [25]; reviewer developed tools; describe the domains assessed: research team, study design, data analysis and interpretations, reporting).	5-6
Appraisal process	Indicate whether the appraisal was conducted independently by more than one reviewer and if consensus was required.	4
Appraisal results	Present results of the quality assessment and indicate which articles, if any, were weighted/excluded based on the assessment and give the rationale.	5 (supplementary file 5).
Data extraction	Indicate which sections of the primary studies were analysed and how were the data extracted from the primary studies? (e.g. all text under the headings "results /conclusions" were extracted electronically and entered into a computer software).	4,5
Software	State the computer software used, if any.	n/a
Number of reviewers	Identify who was involved in coding and analysis.	4
Coding	Describe the process for coding of data (e.g. line by line coding to search for concepts).	5 (supplementary file 4)
Study comparison	Describe how were comparisons made within and across studies (e.g. subsequent studies were coded into pre-existing concepts, and new concepts were created when deemed necessary).	5
Derivation of themes	Explain whether the process of deriving the themes or constructs was inductive or deductive.	13
Quotations	Provide quotations from the primary studies to illustrate themes/constructs, and identify whether the quotations were participant quotations or the author's interpretation	13-20
Synthesis output	Present rich, compelling and useful results that go beyond a summary of the primary studies (e.g. new interpretation, models of evidence, conceptual models, analytical framework, development of a new theory or construct).	20-23

Table S2. Full breakdown of all topics and keyword information

**Type 2 diabetes**	Adherence	UK South Asian	Lifestyle changes	Qualitative
**Glucose**	Help seeking behaviour	UK South Asian Population	Self-monitoring of blood glucose/ Blood glucose monitoring/ Blood glucose level	
**Blood glucose**	Self-care	South Asian	Diet	
**Non-insulin dependant**	Self-management	Bangladeshi	Exercise	
**Non-insulin dependant mellitus**	Patient compliance	Indian	Taking medication	
	Health behaviour	Pakistani	Dietary choices	
	Patient education	Sri Lankan	Dietary foods	
	Patient satisfaction	Nepalese	Nutrition	
	Patient drop-outs	Ethnic minority	Physical activity	
	Physician patient relationships		Fitness	
	Delivery of health care		HbA1c	

Table S3. Database list

**CINAHL**	PUB MED
**PSYCH INFO**	SAGE
**EBOOKCOLLECTION EDUCATION**	SCIENCE DIRECT
**RESEARCH COMPLETE**	JSTORE LIFE SCIENCES
**ERIC**	JSTORE LIFE SCIENCES-I
**REGIONAL BUSINESS NEWS**	OXFORD SCHOLARSHIP ONLINE
**MEDLINE**	HATHI TRUST
**PSYCH ARTICLES**	WEB OF KNOWLEDGE/WEB OF SCIENCE
**SCOPUS**	COGPRINTS
**SOCIAL SCIENCE OPEN ACCESS REPOSITORY (SSOAR)**	

Table S4. Generation of synthetic constructs and the development of a new theoretical framework

COMPONENT	STAGE	DESCRIPTION
Openness	Throughout the meta-synthesis process	Took great care to avoid applying or retaining any pre-conceived notions or theories. Theories were only derived following data analysis.
Analysing immediately	Analysis	The first author completed all data extraction and appraisal. These processes were also conducted and used for comparison by the last (n= 13), second (n= 3) and third (n=3) authors. The research team discussed and reflected on the appraisal of studies, and this information informed aspects of the interpretation within the synthesis.
Coding and comparing	Analysis<br>***Open Coding: the*** Inductive process of breaking down, examining, comparing and categorising the data.<br>***Axial Coding:*** This stage moved from inductive to deductive analysis as the data was assembled in new ways after open coding, by making connections between categories.<br>***Selective coding:*** Process of selecting the core category and the central category around which the final analysis was based and then relating it to all the categories to develop a theory.	Open coding required the application of a comparative method, (e.g. asking questions and making comparisons). Data was primarily broken down by asking simple questions, such as 'what', 'where', 'how' and 'when'. The data was then compared, and similar occurrences were grouped and given the same conceptual label. The process of grouping concepts that occurred most frequently is termed as categorising.<br><br>Each articles findings were read line-by-line. A code was allocated to each line to describe the text. The transcripts were re-read to make comparisons and similarities between the text. The open coding analysis leads to "refining and specifying any borrowed extant concepts" (Strauss & Corbin, 1998).<br><br>Following the open code process, the articles were read once more to identify emerging themes

		from the codes found in the open-code process.
Production of a substantive theory	Analysis and interpretation	Once open, axial and selective coding was completed a CIS framework was developed.

<u>Table S5.</u> Full critical appraisal

Khajuria & Thomas (1992)
Qualitative Framework<br>This study was explore the extent to which traditional beliefs about diet, health and diabetes, as described in the classics of Ayurvedic medicine, are held by Indian (Gujarati) diabetics in Britain.<br>The research question was stated clearly as this study wanted to explore four aims; (1) to identify the areas of possible conflict between traditional remedies and western medicine in the management of diabetes. (2) To explore the knowledge of and use of traditional remedies and dietary modifications in the management of diabetes. (3) To examine the type of foods eaten and the extent to which dietary modifications advocated by western or traditional medical systems are being followed. (4) To examine the dietary advice which patients reported receiving from doctors and/or dietitians, its perceived appropriateness to their eating habits and their satisfaction with this advice.<br>The importance and relevance of this study was stated as the researchers carried out a literature review and a small in-depth was taken prior to carrying out this study. From this it was identified that past research suggests that immigrants to the UK have brought traditional ideas about the role of diet in maintaining health and treating disease and these ideas have not been previously explored.<br>Although this article did not state why qualitative methods were appropriate and chosen they did aim to use an exploratory approach to explore attitudes and experiences. This research seeks to interpret subjective experiences of research participants as they are looking individual experiences.
Study Setting<br>The study setting has not been clearly described. Data was collected through<br>interviews, however, there was no clear indication as to where the interviews took place. It was not clear who was involved in the study. It was indicated that interviews took around 30-40 minutes to complete. It was not clear which philosophical framework the study took, however, a profile search revealed that the authors come from a background of nutrition.<br>This study did not require prolonged engagement or persistent observations in the setting to respond to the research questions. Enough information was provided to assess sufficient depth of data collection and to permit an appreciation of the complexity of the phenomenon.
Study Design<br>The research design was not very clearly described. The researchers justified the research problem clearly but there was no justification as to what methods of analysis where chosen. The sample comprised 28 Gujrati, Hindu, vegetarian diabetic patients. Data was collected through interviews. There was no clear indication of how data was analysed.
Sampling Procedures<br>The sampling selection was briefly described, patients were recruited from two diabetic clinics within London hospitals. The two hospitals were selected to gain different socio-economic background patients. The sampling strategy was not clearly described, though it was stated that an equal number of 14 participants. It was anticipated Hospital A would recruit less affluent and educated patients compared to hospital B. to. Interviews were conducted in Gujrati and English. There was no discussion on how many patients were approached, how many said no and how many chose not to take part. A topic guide was developed for the interviews, The first section consisted of looking at the patients beliefs about the role of diet in the maintenance of health and beliefs about traditional remedies in general. The second section included questions about the patient's condition and the treatment of diabetes, including use of traditional remedies. Two other areas were explored, current eating habits, this was done by using a 24-h recall of all the foods and drinks consumed on the day prior to the hospital appointment and the final areas was personal. There was no information

provided on any events, incidents activities, experiences or social processes which occurred during the course of the study.

Data Collection

The data collection section was very brief, and did not have enough detail. It was stated that tape-recorded semi-structured interviews were conducted, however, it was unclear under what conditions the interviews took place. There was no discussion as to why interviews were chosen as the method of data collection. There was no information on how and under what conditions the research was collected. There was no discussion on triangulation or corroboration for data interpretation. It was not clear who was involved in each stage of the study. There was no reference to an audit trail for data collection, paper trail of field notes, interview transcripts, reflective/administrive journals or memos.

Ethical Issues

No ethical issues were discussed such as informed consent, privacy, and confidentiality and protection from harm. There was no discussion on how informed consent was obtained or a description of the procedure for confidentiality and privacy and how confidentiality and privacy was maintained during and after the study. Ethical adherence has been assumed due to the article being published in the Journal of Human Nutrition and Dietetics. The authors will have confirmed ethical standards prior to publication.

Reflexivity of the Researcher

There was no reflection of any potential bias in aspect of the study. It was unclear who was involved in each stage of the study. There was no indication if the researchers used a reflexive journal for data analysis, prior, during, and after data collection.

Data Analysis

The process of data analysis was not clearly presented there was no description and details on the process of data analysis. There were clear themes but there were no matched quotes from the data. The data had been compiled to match themes. There was no contradictions taken into account and researchers roles where not discussed.

Findings

Although there were no quotes, the results to be explicit and from the experiences participants have faced with type 2 diabetes. There was no indication if member checking was employed. The contribution this study makes is the participants were aware of the importance of diet however, it was not always put into practice. Some reasons where lack of culture specific advice.

Authenticity

No stakeholders were involved in this research.

Fairness

No stakeholders were involved in this research.

Promotion Of Justice

No stakeholders were involved in this research.

Kelleher & Islam (1994)

Qualitative Framework

This study was looking at the problem of integration in Asian people and diabetes. The research question was stated clearly as this study wanted to the aim of this study was to understand and describe ways in which diabetic Bangladeshi people are attempting to integrate traditional and religious rules of eating with the system of modern medicine.

The importance and relevance of this research is that past research has identified that diabetes is prevalent in the Bangladeshi population and although health professionals are aware of this, they did not have much information on their everyday lives and eating habits.

Although this article did not state a clear rationale for using qualitative methods, however, they did aim to explore attitudes and experiences, which points to them using an exploratory approach. This research seeks to interpret subjective experiences of research participants, as they are looking

personal experiences. This study did not clearly state their theoretical framework, a profile search revealed that the authors come from a Sociology background.

Study Setting

Data was collected through interviews and it was stated that they took place in the participant's home. There was no clear rationale as to why they were conducted at the home of the participant, however, due to the nature of the study the setting is appropriate for the research question. It was not clear who was involved in the study, however, it was stated observations were also carried out by the second author a research assistant who was lives in the community. It was indicated that interviews averaged of 1.5 hours. Five participants could not read and write in Bengali and three participants could read and write in English. 30 close family members were also interviewed.

This study did not require prolonged engagement or persistent observations in the setting to respond to the research questions. Enough information was provided to assess sufficient depth of data collection was carried out to permit an appreciation of the complexity of the phenomenon.

Study Design

The research design was not very clearly described. The researchers justified the research problem clearly but there was no justification as to what methods of analysis where chosen. The sample comprised of Bangladeshi patients with type 2 diabetes. Data was collected through interviews. It was stated that this research paper was preliminary analysis, however, there was no clear indication of how data was analysed.

Sampling Procedures

The sampling selection was briefly described, patients were recruited from one health centre in the Tower Hamlets district but did not state which health centres. The sampling strategy was clearly described as it was stated that to achieve representativeness by a quota sample of 25 men and 15 women of varying ages. Of the 20 people, 12 were men and eight were women. Eight were under 50 years old and 12 were over 50, they did state that there difficult to establish the exact age of some of the older men. The sample consisted of people who had come from village life in Bangladesh to live in the East End of London. None of the women were employed, out of the 12 men, one was employed, one was a student and 10 were not working. Five participants could not read and write in Bengali; three could read and write in English. Two interviews were conducted in the Sylheti dialect. There was no discussion on how many patients were approached, how many said no and how many chose not to take part. There was no information to suggest if the interview was guided by a topic guide. There was no information provided on any events, incidents activities, experiences or social processes which occurred during the course of the study.

Data Collection

The data collection section was very brief, and did not have enough detail. It was stated that tape-recorded interviews were conducted, however, it was unclear if it was structured or semi-structured interviews. There was no discussion as to why interviews were chosen as the method of data collection. There was further information about the observations which were carried out from the second author. There was no information on how and under what conditions the research was collected. There was no discussion on triangulation or corroboration for data interpretation. It was not clear who was involved in each stage of the study. There was no reference to an audit trail for data collection, paper trail of field notes, interview transcripts, reflective/administrive journals or memos.

Ethical Issues

No ethical issues were discussed such as informed consent, privacy, and confidentiality and protection from harm. There was no discussion on how informed consent was obtained or a description of the procedure for confidentiality and privacy and how confidentiality and privacy was maintained during and after the study. Ethical adherence has been assumed due to the article being published in the Journal of the Royal Society of Medicine. The authors will have confirmed ethical standards prior to publication.

Reflexivity of the Researcher

There was no reflection of any potential bias in aspect of the study. It was unclear who was involved in each stage of the study. The second author who was the research assistant lived within the community, it was not clear if the second author had any input in sample recruitment and choice of location. There was no reflection of any possible bias due to the second author living within the community under study. There was no indication if the researchers used a reflexive journal for data analysis, prior, during, and after data collection.

Data Analysis

The process of data analysis was not clearly presented as it mentions initially that 40 participants were interviewed and preliminary analysis was conducted based on 20 of the participants but there was no justification as to how the 20 participants were selected. There was no description and details on the process of data analysis. There were no clear themes but the results were matched with quotes from the data. There was no contradictions taken into account and researchers roles where not discussed.

Findings

Although there were no themes, the quotes appear to be explicit and from the experiences participants have faced with type 2 diabetes. There was no indication if member checking was employed. Examples from the text were used to support conclusions, however there were no clear themes identified. The contribution this study makes is that it identifies the need for self-help groups to be available as this will help the integration of the management of diabetes in everyday lives.

Authenticity

No stakeholders were involved in this research.

Fairness

No stakeholders were involved in this research.

Promotion Of Justice

No stakeholders were involved in this research.

Chowdhury et al (2000)

Qualitative Framework

This study explored informants' health beliefs and illness maps of diabetes as well as their specific beliefs and behaviours about food. The researchers identified that to promote `healthier' food choices they first need to understand how individuals select the foods they eat and foods they choose not to eat. The purpose of this study was to explore the food beliefs and classification system of British Bangladeshis with diabetes. The study used a variety of qualitative methods to explore health beliefs of the participants.

Although this article did not state a clear rationale for using qualitative methods, however, they did state that the aim was to explore attitudes and experiences, which points to them using an exploratory approach. This research seeks to interpret subjective experiences of research participants, as they are looking personal experiences. This study demonstrated the use of principles of anthropology, specifically, 'within any cultural group there is considerable variation and idiosyncrasy'.

Study Setting

The first author collected data at the participant's home. There was a lengthy process of data collection as a range of methodological approaches were used in order to collect comprehensive data, such as audiotaped narrative, semi structured interviews, focus group discussions, construction of family tree, pile sorting exercise where disease ranking, foods, meal menus, "preferred" and "healthy" body size were ranked, structured vignette method, feedback of preliminary constructs to focus groups and study of patients general practice case notes. There was no information if the first author collected all the data on his own, or if there was anybody else involved. There was no indication on how long each of the methodological approaches lasted.

This study did not require prolonged engagement or persistent observations in the setting to respond to the research questions. Enough information was provided to assess sufficient depth of data collection was carried out to permit an appreciation of the complexity of the phenomenon.

Study Design

The research design was clearly described. Informants were asked to describe their eating regimen and what they had eaten during the previous day. In addition, they were asked to sort particular food items into `permitted' , `prohibited' and `neither permitted nor prohibited' , and again into `good for my health' , `bad for my health' and `neither good nor bad' . Explanations were sought in the case of discrepancies between the two sources of information. As part of their norms of hospitality, informants frequently offered the researcher *nasta* (snacks) or *bhath* (main meal), which allowed further discussions about food choices, mode of cooking, use of ingredients. The classification of particular food items was used as the basis for tape recorded individual interviews and focus group discussions in which the basic classificatory schemas were explored in depth. The sample comprised of Bangladeshi patients with type 2 diabetes. The first author A.Mu'Min Chowdhury conducted the interviews in the participants homes. It was not clear, where the other data was collected and if anthropological analysis was used to analyse all the data as anthropological principles were applied to the study.

Sampling Procedures

The sampling selection was briefly described, this study recruited 40 participants from a primary care setting in the inner city areas of Tower Hamlets, Newham or Islington in London. An equal mix of genders were recruited aged between 24 and 78, and were first-generation migrants. There was no indication as to which sampling strategy was used. The recruitment was through practice diabetes registers or manual note search. They did clarify that they have not utilised a representative sample for the Bangladeshi community as a whole just a small sector of the society.

Data Collection

The data collection section was described. Participants were asked to describe their eating routine and what they had eaten during the previous day. They were also asked to complete a sorting task were particular food items were shown and they had to put into the pile of `permitted, 'prohibited' and `neither permitted nor prohibited' , and again into `good for my health' , `bad for my health' and `neither good nor bad'. Explanations were sought in the case of discrepancies between the two sources of information. The task was used a starting point to guide the tape recorded individual interviews and focus group discussions. Preliminary impressions were fed back to informants in a second round of interviews and focus groups to validate the constructs. There was no discussion on triangulation or corroboration for data interpretation. It was not clear who was involved in each stage of the study. There was no reference to an audit trail for data collection, paper trail of field notes, interview transcripts, reflective/administrive journals or memos.

Ethical Issues

No ethical issues were discussed such as informed consent, privacy, and confidentiality and protection from harm. There was no discussion on how informed consent was obtained or a description of the procedure for confidentiality and privacy and how confidentiality and privacy was maintained during and after the study. Ethical adherence has been assumed due to the article being published in the Anthropology and Medicine. The authors will have confirmed ethical standards prior to publication.

Reflexivity of the Researcher

The first author AMC was a male Bangladeshi social anthropologist who spoke the Bangladeshi mother tongue Sylheti. He resided and worked in London for over 10 years. His previous studies were focused on kinship networks in rural Bangladesh. The first authors role was clearly described however, there was no reflection regarding any influence bias he may have had on the participants.

Data Analysis<br>The process of data analysis was not presented in detail. NUDIST (N6, 2002) was used to analyse the data. There was no description of the analysis. There were sufficient findings as they were able to gather a number of themes from the data, however there were no quotes from the participants to match the themes. There was no contradictions taken into account and researchers roles where not discussed. There was no contradictions taken into account and all the researchers roles where not discussed.
Findings<br>The researchers have discussed credibility of their findings by looking at past research and comparing and contrasting their findings. They have identified many areas future research can look into and provided a detailed and a clear conclusion as to what they believe the research has found. Member checking was employed as preliminary impressions were fed back to informants in a second round of interviews and focus groups to validate the constructs.<br>The researchers provide thick description of the sample and results. The main findings indicated that dietary advice should reflect religious restrictions, ethnic customs and the different cultural meaning of particular foods, while also acknowledging the ability of the individual to exercise choice within those broad limits.
Authenticity<br>No stakeholders were involved in this research.
Fairness<br>No stakeholders were involved in this research.
Promotion Of Justice<br>No stakeholders were involved in this research.
**Bissell et al (2004)**
Qualitative Framework<br>The purpose and research question is clearly stated as the researchers seek to empirically explore the relevance of a re-framed consultation through qualitative interviews with a small group of English speaking patients of Pakistani origin with a diagnosis of type 2 diabetes. The goal of this research was to explore Pakistani group's experience of coping with diabetes and its treatment, and explore how they spoke about their relationships with health care professionals. The authors state that the importance and relevance of this study is that although there is a lot of research on adherence and concordance, the findings always conclude that people do not always follow the doctor's orders, when in fact more research needs to be conducted on the models of health care to find better ways to develop the doctor patient relationship. A qualitative approach was appropriate to answer the research question as they stated that the aim of the study was to explore the meanings and understandings of patients in depth which seeks to interpret subjective experiences of research participants. It was not clear which philosophical framework the study took, however, a profile search revealed that the authors come from a background of public health.
Study Setting<br>The setting of the study was appropriate and specific for exploring the research question as it was clear that the interviews took place at the participant's home and the first author conducted the interviews, however, length of time the interviews took was not stated. There was no clear rationale as to why they were conducted at the home of the participant, however, due to the nature of the study the setting is appropriate for the research question.<br>This study did not require prolonged engagement or persistent observations in the setting to respond to the research questions. Enough information was provided to assess sufficient depth of data collection was carried out to permit an appreciation of the complexity of the phenomenon.

Study Design<br>The research design was a qualitative design as the study aimed to explore the meanings and understandings of patients in depth. The nature of the sample focussed on a specific group being from the Pakistani origin. Data was collected through interviews with only english speaking participants. Grounded theory (Glaser&Strauss, 1967) was used for analysis. The research design was made clear as it was noted that qualitative approach was used as the aim was to explore meanings and understanding in depth.
Sampling Procedures<br>Recruitment was from two primary and one secondary care diabetes centre in the North West of England. The recruitment process consisted of health professionals asking patients who fit the eligibility criteria if they would be willing to take part in the study, if they agreed they either contacted the researcher or their details were passed onto the researcher. From this 16 participants were recruited, however, the health professionals did not note how many people refused to take part. Another six participants were recruited through snowball sampling were the interviewees were asked if they knew if any family, friends from the local community would be willing to take part. There were a total of 21 participants. As grounded theory was used the sample size depended on reaching saturation of data. There was no information to suggest if the interview was guided by a topic guide. There was no information provided on any events, incidents activities, experiences or social processes which occurred during the course of the study.
Data Collection<br>Data was collected through interviews. There was no indication if the researcher used a topic guide or prompt questions. The data was tape recorded and adhered to by (Glaser and Strauss, 1967) grounded theory protocol.<br>There was no indication if a variety of methods from triangulation took place, however, it was identified that due to the grounded theory protocol the categories the first author produced were verified by the second author. There was no reference to an audit trail for data collection, paper trail of field notes, interview transcripts, reflective/administrive journals or memos.
Ethical Issues<br>No ethical issues were discussed such as informed consent, privacy, and confidentiality and protection from harm. There was no discussion on how informed consent was obtained or a description of the procedure for confidentiality and privacy and how confidentiality and privacy was maintained during and after the study. Ethical adherence has been assumed due to the article being published in the Journal of Social Sciences & Medicine. The authors will have confirmed ethical standards prior to publication.
Reflexivity of the Researcher<br>The researchers identified potential and actual biases in the data collection phase when conducting interviews by providing enough description of the background and the role in the research design. There was no information provided if the researcher's integrated the use of a reflexive journal in the data analysis and interpretation prior, during and after data collection.
Data Analysis<br>The process of data analysis was presented with detail to provide an insight into the meanings and perceptions of the sample, there was enough description to allow the reader to assess what the data analysis was based on and was consistent with the method and purpose of the study. The researcher's explains how the data was presented and how they selected the findings from the sample to demonstrate the analysis process by providing extracts of the transcripts. There was sufficient data was presented to support findings. All quotes were used to match concepts and themes derived from the raw data.

Findings<br>The findings emerge from the experiences of the sample and findings are explicit. There was adequate discussion of the evidence both for and against the researcher's arguments as they discussed their findings even though they were not consistent with the literature out there. The researchers also discussed their findings in relation to the original research question. They discussed how the themes emerged from the experiences of the participants but there was no discussion on how the themes emerged from the influences of the researcher's.<br>Member checking was not employed as participants were not asked to confirm or disconfirm the accuracy of the researcher's observations and interpretations.<br>The researchers provide a thick description of the sample and results to appraise transferability by providing sufficient details about the context to assess whether the findings emerged from the data. There was no discussion if the findings might be applied to samples in similar settings. Participants included in the study and quotes were used from the text to support conclusions. The researcher's discusses the contributions the study makes to the current relevant based literature and states that patient involvement has a positive effect on patient's lives.
Authenticity<br>No stakeholders were involved in this research.
Fairness<br>No stakeholders were involved in this research.
Promotion Of Justice<br>No stakeholders were involved in this research.
**Lawton et al (2005)**
Qualitative Framework<br>The purpose and research question was to explore British Pakistani and British Indian patients' perceptions and experiences of taking oral hypoglycaemic agents (OHAs).The objective was to inform their future care by developing an intervention that is delivered in a cultural sensitive approach. The importance and relevance of this study was to explore an unstudied area—the attitudes of Pakistani and Indian patients with type 2 diabetes towards, and experiences of, taking OHAs. This research seeks to interpret subjective experiences of research participants. It was not clear which philosophical framework the study took, however, a profile search revealed that the authors come from a background of health science.
Study Setting<br>The setting of the study was appropriate and specific for exploring the research question as it was clear that the interviews took place at the participant's home and the second author conducted the interviews in English and Punjabi, the interviews lasted on average 1 hour. There was no clear rationale as to why they were conducted at the home of the participant, however, due to the nature of the study the setting is appropriate for the research question.<br>This study did not require prolonged engagement or persistent observations in the setting to respond to the research questions. Enough information was provided to assess sufficient depth of data collection was carried out to permit an appreciation of the complexity of the phenomenon.
Study Design<br>Qualitative methods were used to inspire participants to demonstrate their own understandings and meanings. Participants were selected on the basis of being Pakistani or India as Scotland has a high population of this culture. Topics covered in this study where 1) current and past diabetes treatment regimens, 2) experiences of taking oral hypoglycaemic agents (OHAs) and other drugs (including perceptions and understandings of symptoms and side effects), 3) perceived efficacy of OHAs and other drugs (including complementary therapies) in the short term and long term, 4) commitment to adhering to OHAs and others regimens; changes in commitment over time, 5) experiences of health care and health professionals in Britain and abroad, 6) perceptions of past, present, and future health. Strauss and Corbin (1990) grounded theory framework was used to

analyse the data. The data collection method was appropriate as interviews were guided around the topics under study.

Sampling Procedures

Recruitment was carried out by health professionals. The participants were purposively selected from five general practises in Edinburgh with high Indian and Pakistani patients, purposeful sample was used to achieve a representative sample. The sample was narrowed down to Indians and Pakistanis and excluded all other South Asian descents due to logistical problems and small sample of residents in Scotland. The sample was selected on the basis of gender, religious and/or ethnic group, gender and duration of diabetes. The study recruited 32 participants which consisted of Pakistanis (n=23) and Indians (n=9), 15 males and 17 females. It was illustrated that notes were made during regular meetings which was discussed in the initial framework which help develop the themes and subthemes. There was no information provided on any events, incidents activities, experiences or social processes which occurred during the course of the study.

Data Collection

Interviews were taped recorded and conducted using topic guides by the second author. Interviews were conducted in either Punjabi or English. Interviews took place until saturation of data according to the grounded theory guidelines. Data was interpreted by the researcher and two translators to ensure triangulation. Transcripts were read and compared across a team and were discussed during meetings and notes were taken during the meetings. NUD*IST a qualitative package was used to facilitate data coding and retrieval. There was no reference to an audit trail for data collection, paper trail of field notes, reflective/administrative journals or memos.

Ethical Issues

The Lothian Research Ethics Committee approved the study. Some ethical issues were discussed such as confidentiality was assured to the participants and interviews were conducted in a sensitive and non-judgmental manner. The article was published in the BMJ, the authors will have confirmed ethical standards prior to publication.

Reflexivity of the Researcher

There was no discussion if the researcher identified potential and actual biases in the research, such as the when formulating the question. There was no reflection of the data collection, including sample recruitment and choice of location. The interviews were carried out by the second author who was bi-lingual carried out, however, there was no information provided that allows us to see if there was any potential influence or bias on the study. They also stated that they got health professionals to recruit participants as past research has found it difficult to recruit participants from ethnic minority backgrounds.

Data Analysis

Data was analysed using the grounded theory. The guidelines for this approach were followed and data analysis was presented with sufficient detail and depth to provide insight into the meanings and perceptions of the sample. Quotes were used to match themes derived from the data. To reduce bias two translators also analysed the transcripts as well as the researcher.

Findings

The findings emerged from the experiences of the participant's perceptions and experiences of taking oral hypoglycaemic agents and were discussed by providing example of the transcripts to back the themes that had been derived from the data. Member checking was employed some respondents were contacted after the interviews to clarify issues and validate findings. There was a description of the sample and results to appraise transferability. The researchers discussed how the study contributes to the understanding of the cohort under study. The findings of this study indicate the importance of taking into consideration cultural factors to ensure patients are given appropriate advice.

Authenticity

No stakeholders were involved in this research.

Fairness

No stakeholders were involved in this research.
<u>Promotion Of Justice</u><br>No stakeholders were involved in this research.
**Macden & Clarke(2006)**
<u>Qualitative Framework</u><br>This study aimed to report knowledge of the experiences of South Asian people with diabetes in the UK in relation to socio-cultural and dietary practices, religion and ageing influences on the perception and understanding of risks. The research question was stated clearly as this study wanted to explore socio-cultural and dietary practices, religion and ageing influences on the perception and understanding of risks. The importance and relevance of this study was stated as the researchers identified that past research suggesting the need for the study. Although this article did not state why qualitative methods were appropriate and chosen they did aim to explore experiences which indicates them using exploratory approach in qualitative methods. This research seeks to interpret subjective experiences of research participants as they are looking individual experiences. It was not clear which philosophical framework the study took, however, a profile search revealed that the authors come from a background of nursing.
<u>Study Setting</u><br>The study setting has not been clearly described. Data was collected through focus group interviews, however, there was no clear indication as to where interviews took place. There was no clear rationale as to why this method was chosen. It was not clear who was involved in the study, however, it was noted that ethnic health development workers where used interpreters with non-English speaking participant's. There was no indication as to how long the focus group interviews lasted.<br>This study did not require prolonged engagement or persistent observations in the setting to respond to the research questions. Enough information was provided to assess sufficient depth of data collection was carried out to permit an appreciation of the complexity of the phenomenon.
<u>Study Design</u><br>The researchers justified the research problem clearly, however, the research design was not very clearly described. The sample comprised of ethnic health development workers and individual interviews with 20 older people with diabetes. The number of ethnic health development that took part was not clear. Data was collected through focus group interviews and analysed using grounded theory methodology. There was no indication if a topic guide was used.
<u>Sampling Procedures</u><br>The sampling selection was not clear, it was briefly stated that 20 South Asian men and women with type 2 diabetes who are living in NE England. Representations were sought from non-English speaking groups (comprising eight of those interviewed) and the Bangladeshi community (five of those interviewed). The sampling strategy was not stated. There was no information provided on any events, incidents activities, experiences or social processes which occurred during the course of the study.
<u>Data Collection</u><br>The data collection section was very brief, and did not have enough detail. It was stated that tape-recorded interviews were conducted. There was no discussion as to why interviews were chosen as the method of data collection. There was no information on how and under what conditions the research was collected. Issues related to validity and rigour were addressed using triangulation at two levels (data and interdisciplinary), reflexivity, peer debriefing and maintaining an audit trail. Data was analysed using NVIVO 2, a Qualitative software package, and this paper reports some of the initial open coding. It was not clear who was involved in each stage of the study. There was no reference to a, paper trail of field notes, interview transcripts, reflective/administrive journals or memos.

Ethical Issues<br>It was briefly stated that ethical and governance approval was secured from the appropriate organizations. No other ethical issues where discussed. The article was published in the International Journal of Older People Nursing the authors will have confirmed ethical standards prior to publication.
Reflexivity of the Researcher<br>There was no discussion if the researcher identified potential and actual biases in the research, such as the when formulating the question. There was no reflection of the data collection, including sample recruitment and choice of location.
Data Analysis<br>Data was analysed using the grounded theory. However the process for this approach were not discussed. Quotes were used to match themes derived from the data.
Findings<br>The findings emerged from the experiences of the experiences of South Asian people with diabetes in the UK in relation to socio-cultural and dietary practices, religion and ageing influences on the perception and understanding of risks. They were discussed by providing example of the transcripts to back the themes that had been derived from the data. Although not clearly discussed member checking was employed as participants where approached for validation wherever possible.. There was no clear description of the sample and but the results enables us to appraise transferability. The researchers discussed how the study contributes to the understanding of the cohort under study. The findings of this study indicate how risk perception in older South Asian people is influenced primarily by their understanding of the disease, perceptions of its severity, ageing, dietary practices and religion.
Authenticity<br>No stakeholders were involved in this research.
Fairness<br>No stakeholders were involved in this research.
Promotion Of Justice<br>No stakeholders were involved in this research.
**Lawton et al (2006)**
Qualitative Framework<br>The purpose and research question of this study was to explore patients' perceptions and experiences of undertaking physical activity as part of their diabetes care which is stated clearly in the abstract. The goal of the research was to inform Pakistani and Indian patients' future diabetes care. They stated the importance and relevance by discussing how taking part in physical activity is very low in this specific cohorts and there needs to be an exploration of their experiences of physical activity. A qualitative approach was considered to be appropriate to answer the research question as this would allow themes to be identified and explored during the study. It was not clear which philosophical framework the study took, however, a profile search revealed that the authors come from a background of health science.
Study Setting<br>Interviews were conducted in the participant's home by the second author who was a bi-lingual author as the interviews were conducted in either Punjabi (n=19), English (n=9) or combination of Punjabi and English (n=4). The interviews took around 1 hour. There was no indication if there was a history of involvement with the groups under study to build trust and comfort. However, the data collection process appeared to be appropriate to meet the needs of the study.<br>This study did not require prolonged engagement or persistent observations in the setting to respond to the research questions. Enough information was provided to assess sufficient depth of data collection was carried out to permit an appreciation of the complexity of the phenomenon.

Study Design<br>As the researchers justified and discussed why they wanted to use qualitative research as they wanted participants to display their own understandings and meanings, and to raise and discuss issues they perceived as relevant. The nature of the sample was appropriate as they recruited a sample of Indian and Pakistanis and Bangladeshi participants were excluded due to being a hard to reach group. The data collection method was appropriate as interviews could be guided around the topics under study. The method of analysis was clearly stated that grounded theory (Glaser&Strauss, 1967) was chosen as the analysis.
Sampling Procedures<br>Recruitment took place from 5 general practises in Edinburgh with high Indian and Pakistani patients. It was identified that recruiting samples from ethnic minority groups can be time consuming and there is difficulties in accessing participants from this group, for this reason clinicians wrote to patients in languages which they could understand asking them to 'opt in' and snowball sampling was used in local communities for recruitment. Purposeful sampling was used in the recruitment process as age, length of diagnosis, fluency of English and gender established if the individual could participate to ensue diversity in participants. Through these procedures 32 participants were recruited, which included Ethnic origin: Pakistani (n=23) and Indian (n=9), Religion: Muslim (n=22), Hindu (n=4), Sikh (n=4) and Christian (n=1) Recruitment stopped in line with the grounded theory approach when no new themes emerged from the interviews. It was illustrated that notes were made during regular meetings which was discussed in the initial framework which help develop the themes and subthemes. There was no information provided on any events, incidents activities, experiences or social processes which occurred during the course of the study.
Data Collection<br>Data was collected at the participant's home by conducting interviews which were tape recorded and guided by a topic guide which was initially developed by conducting a literature review after piloting the questions, the guide was revised by the researchers with the input of health professionals. Data was collected until saturation of themes. Interviews were offered in English, Punjabi, Urdu and Hindi. The second author carried out the interviews. The interviews were carried out in Punjabi (n=19), Punjabi and English (n=9) and English (n=4). Triangulation was taken into consideration as by the transcripts and recordings being checked by translators who speak Pakistani and Indian alongside the researcher. Data was conducted and analysed by the second author, but analysis was constantly reviewed by the research team. NUD*IST (Boyatzis, 1998) a qualitative package was used to facilitate data coding and retrieval. There was no reference to an audit trail for data collection, paper trail of field notes, reflective/administrive journals or memos.
Ethical Issues<br>There was statement which stated that informed consent was obtained when the interviews commenced and the Lothian Research Ethics committee approved the study. Although ethical issues were not adequately discussed the authors will have confirmed ethical standards prior to publication. The article was published in the Health Education Research.
Reflexivity of the Researcher<br>The researcher identified potential and actual biases by conducting a literature review to gain a wider understanding about the research topic and based the interviews on aspects which have been understudied. The second author carried out the interviews, however there was no information provided that allows us to see if there was any influence on the results. They also stated that they got health professionals to recruit participants as past research has found it difficult to recruit participants from ethnic minority backgrounds.

Data Analysis<br>The process of data analysis and a description of the analysis were presented with sufficient detail and depth to provide insight into the meanings and perceptions of the sample as grounded theory was used was stated that emerging themes were explored with participants. Sufficient data was presented to support findings. There was no indication if contradictory data are taken into account, however researchers reviewed the finding and discussed results on a regular basis. Quotes from the interview were used to match concepts and themes derived from the raw data. There was no indication as to which author conducted the analysis.
Findings<br>The findings emerged from the experiences of the sample and were explicit. The themes emerged from the experiences of the participants as the researchers discussed the results in-depth in the discussion section. Member checking was not employed as participants were not asked to confirm or disconfirm the accuracy of the researcher's observations and interpretations or asked their views about the credibility of the findings and interpretations. There was a discussion of how the findings emerged from the data as examples from the text were used to support conclusions. This study identified that cultural appropriate ways need to be taken into account to promote physical activity.
Authenticity<br>No stakeholders were involved in this research.
Fairness<br>No stakeholders were involved in this research.
Promotion Of Justice<br>No stakeholders were involved in this research.
**Lawton et al (2008)**
Qualitative Framework<br>The purpose and research question was to look at food and eating practices from the perspectives of Pakistanis and Indians with type 2 diabetes, their perceptions of the barriers and facilitators to dietary change, and the social and cultural factors informing their accounts. The objective was to inform the delivery of dietary advice for those affected by the disease. The importance and relevance of this study is that there was more quantitative research on this topic and there was a need to adapt qualitative research which would allow the researchers to gain in-depth understanding on dietary habits as the population under study has been found to have bad dietary habits which cause complications in their diabetes. This research seeks to interpret subjective experiences of research participants. It was not clear which philosophical framework the study took, however, a profile search revealed that the authors come from a background of health science.
Study Setting<br>The interviews took place in the participant's home by a bilingual researcher who spoke English and Punjabi and lasted around 1 hour. The interviews were conducted to gain an in-depth insight into the topic under study, the interviews would shed light to issues that can be missed if quantitative methods such as questionnaires were distributed.<br>This study did not require prolonged engagement or persistent observations in the setting to respond to the research questions. Enough information was provided to assess sufficient depth of data collection was carried out to permit an appreciation<br>of the complexity of the phenomenon.

Study Design<br>Qualitative methods were used as the researchers identified that the topic of diet has mostly been researched upon using quantitative methods which does not take into social and cultural which can be best captured by using qualitative methods as interviews will allow gaining a deeper insight as to the social and cultural factors that influence diet. Participants were selected on the basis of being Pakistani or India as Scotland has a high population of this culture. Topics covered in this study where food and eating practices during a typical day and on special occasions; changes/continuities in one's diet since migration (if relevant) and diagnosis of type 2 diabetes; perceived barriers and facilitators to dietary change; role of self and others in food purchase and preparation; perceptions of the relationship between diet and health; social and symbolic role of food in everyday life; and (in light of emerging findings) perceived impact of diet and dietary changes on self and identity. Strauss and Corbin (1990) grounded theory framework was used to analyse the data. The data collection method was appropriate as interviews could be guided around the topics under study. The method of analysis was clearly stated that grounded theory (Glaser&Strauss, 1967) was chosen as the analysis.
Sampling Procedures<br>Recruitment was carried out by health professionals. The participants were purposively selected from five general practises in Edinburgh with high Indian and Pakistani patients, purposeful sample was used to achieve a representative sample. The sample was narrowed down to Indians and Pakistanis and excluded all other South Asian descents due to logistical problems and small sample of residents in Scotland. The sample was selected on the basis of gender, religious and/or ethnic group, gender and duration of diabetes. The study recruited 32 participants which consisted of Pakistanis (n=23) (Muslims (n=22, Christian n=1)) and Indians (n=9) (Hindus (n =4), Sikhs (n=5)), 15 males and 17 females. It was illustrated that notes were made during regular meetings which was discussed in the initial framework which help develop the themes and subthemes. There was no information provided on any events, incidents activities, experiences or social processes which occurred during the course of the study.
Data Collection<br>Interviews were taped recorded and conducted using topic guides by the second author. Interviews were conducted in either Punjabi or English. Interviews took place until saturation of data according to the grounded theory guidelines. Data was interpreted by the researcher and two translators to ensure triangulation. Transcripts were read and compared across a team and were discussed during meetings and notes were taken during the meetings. NUD*IST a qualitative package was used to facilitate data coding and retrieval. There was no reference to an audit trail for data collection, paper trail of field notes, reflective/administrive journals or memos.
Ethical Issues<br>The Lothian Research Ethics Committee approved the study. Although all ethical issues were not adequately discussed the authors will have confirmed ethical standards prior to publication, The article was published in the Health & Ethnicity Journal authors will have confirmed ethical standards prior to publication.
Reflexivity of the Researcher<br>There was no discussion if the researcher identified potential and actual biases in the research, such as the when formulating the question. There was no reflection of the data collection, including sample recruitment and choice of location. The second author carried out the interviews, however there was no information provided that allows us to see if there was any influence on the results. They also stated that they got health professionals to recruit participants as past research has found it difficult to recruit participants from ethnic minority backgrounds.
Data Analysis<br>Data was analysed using the grounded theory. The guidelines for this approach were followed and data analysis was presented with sufficient detail and depth to provide insight into the meanings

and perceptions of the sample. Quotes were used to match themes derived from the data. To reduce bias two translators also analysed the transcripts as well as the researcher.

Findings

The findings emerged from the experiences of the participant's diet and were discussed by providing example of the transcripts to back the themes that had been derived from the data. There was no indication if participants were asked to confirm or disconfirm the accuracy of the researcher's observations and interpretations. There was a description of the sample and results to appraise transferability. The researchers discussed how the study contributes to the understanding of the cohort under study. The findings of this study indicate the importance of promoting dietary changes which work with the kinds of foods Indians and Pakistanis are eating already. Future research needs to look at what health professional's perceptions and understandings of the Pakistani and Indian diet.

Authenticity

No stakeholders were involved in this research.

Fairness

No stakeholders were involved in this research.

Promotion Of Justice

No stakeholders were involved in this research.

Choudhury et al (2009)

Qualitative Framework

The purpose and research question of this study was to examine the understanding and beliefs of people with diabetes from the Bangladeshi community living in the UK. The goal of this research was to investigate the understanding and beliefs of Bangladeshi people about the condition, its causes, prevention, and management. This study claimed that it is important and relevant as they found very little evidence base on ethnic groups views and beliefs about diabetes. It was not clear which philosophical framework the study took, however, a profile search revealed that the authors come from a background of Public health.

Study Setting

The participants were invited to a peer educational programme where semi structured interviews took place on a one-to-one basis in English or Sylheti. The length of time in the setting was 1 -1.5 hours. There was no clear rationale as to why interviews where used, however, due to the nature of the study the setting is appropriate for the research question. It was not clear who was involved in the study, however, it was stated that the interviews were conducted by a bi-lingual researcher. Due to time constraint interviews where capped at 14 Bangladeshi participants. This study did not require prolonged engagement or persistent observations in the setting to respond to the research questions. Enough information was provided to assess sufficient depth of data collection was carried out to permit an appreciation of the complexity of the phenomenon.

Study Design

The research design was appropriate for the research question as the research looked at an intervention that the participants attend which would be suitable for a qualitative design. The nature of the sample was Bangladeshi patients with type 2 diabetes, data was collected through interviews. The method of analysis was not clear but the write-up of the analysis suggests an analysis where themes emerge may have been used.

Sampling Procedures

The recruitment was carried out through posters, word of mouth, asking local members of the community to part take and asking local mosque to make announcements through radio transmitters. There was no specific sampling strategy used. From the 14 participants, four participants were male, and ages ranged from 26-67 and diabetes duration five of the participants had diabetes for five years, two participants had it for 5-10 years, four participants had it 10-15 years and three participants had it for 15+ years. There was no discussion on recruitment issues. The

interviews where guided with a structured interview was schedule. There was no information provided on any events, incidents activities, experiences or social processes which occurred during the course of the study.
Data Collection<br>It was clear that data was collected through structured interviews and was audiotaped. There was no discussion as to why interviews were chosen as the method of data collection. For triangulation two researchers transcribed and analysed the results. There was no indication on who collected the data and who was involved in each phase of the study. The data was collected between September 2007-February 2008. There was no reference to an audit trail for data collection, paper trail of field notes, reflective/administrive journals or memos.
Ethical Issues<br>There was a brief mention that consent was asked before the interview took place and that an ethical approval was granted from the Multicentre Research Ethics Committee (MRES) for wale. The article was published in the Diabetic Medicine Journal, although ethical issues were not adequately discussed the authors will have confirmed ethical standards of the prior to publication.
Reflexivity of the Researcher<br>The researcher did not identify potential and actual biases in the formulation of the research question, data collection, including sample recruitment and choice of location, initial expectations of study results, understanding of their roles within the production of knowledge. There is a brief mention that the researcher was bilingual (English and Sylheti) but there was no discussion on how that may influence the results. There no use of reflexive journals during and after data collection.
Data Analysis<br>This section was not comprehensive as there was no explanation on how the data was presented from the original sample to demonstrate the analysis process. There was no indication whether the researcher's roles influenced analysis and selection of data for presentation. Quotes were used to match concepts and themes derived from the raw data. Discrepancies and new themes were discussed during the analysis.
Findings<br>The findings emerge from the experiences of the sample. There was no adequate discussion of evidence both for and against the researcher's arguments. The researcher's discussed the credibility of their findings by reflecting on articles. Findings were discussed in relation to the original research question and it was clear that the themes emerged from the experiences of the participants from the structured interviews.<br>Member checking was not employed as participants were not asked to confirm or disconfirm the accuracy of the researcher's observations and interpretations or solicit their views about the credibility of the findings and interpretations<br>Examples from the interview were used to support conclusions. The researcher discusses contributions the study makes to existing understanding as discuss their findings with relevant based literature. The study concluded that there is a need for improve information for Bangladeshi people with type 2 diabetes and much of this information might need to come from health professionals.
Authenticity<br>No stakeholders were involved in this research.
Fairness<br>No stakeholders were involved in this research.
Promotion Of Justice<br>No stakeholders were involved in this research.
**Macden & Clarke(2010)**

Qualitative Framework<br>This study's aim was to analyse risk perception among older South Asian people with Type 2 Diabetes in the UK. The research question was stated clearly as this study wanted to. This study focuses on the findings that were relevant to the Locus Of Control (LOC) influencing risk perception in the population studied, The importance and relevance of this study was stated as the researchers identified that past research suggesting the need for the study. This article stated why qualitative methods were appropriate as the research aims were most appropriately addressed through a qualitative design which draws on the concepts underpinning LOC. This research seeks to interpret subjective experiences of research participants as they are looking individual experiences. . It was not clear which philosophical framework the study took, however, a profile search revealed that the authors come from a background of nursing.
Study Setting<br>The study setting has not been clearly described. Data was collected through focus group interviews, however, there was no clear indication as to where interviews took place. The study used a qualitative research design, using grounded theory with its theoretical foundations drawn from Symbolic Interactionism. There was no indication as to how long the focus group interviews lasted. The first author (LM) prepared the study design, data analysis and manuscript.<br>This study did not require prolonged engagement or persistent observations in the setting to respond to the research questions. Enough information was provided to assess sufficient depth of data collection was carried out to permit an appreciation of the complexity of the phenomenon.
Study Design<br>The researchers justified the research problem clearly and described adequately. Data was collected between March 2004– February 2005. Six health development workers representing ethnic minorities took part in a focus group interview (three female and three male health development workers were bilingual and from South Asia India, Bangladesh or Pakistan). Seven individual interviews with healthcare professionals (three physicians, three nurse specialists and a dietitian).<br>Twenty interviews were conducted with South Asian men and women who were over 60 years of age, diagnosed with Type 2 diabetes and lived in the North East of England.
Sampling Procedures<br>Participation was sought from both non-English-speaking and English-speaking South Asians. A representative sample was trageted from three major South Asian communities in the UK (Bangladesh, India and Pakistan). Twelve participants belonged to the Muslim religion. The health development workers were used as interpreters while interviewing non-English-speaking participants. Cross Language Interpretation process (Larson 1998) was used for the methodological challenges within the language barriers in this study. The interpreter translated the vocabulary and grammatical structure of the words taking into account the individual situation and the overall cultural context of the language. Nine interviews were carried out using this method with the non-English-speaking participants in this study (seven women and two men).<br>The sampling strategy was through the health development workers and Diabetes nurse specialists representing ethnic minorities. In an attempt to maximize the response rate, links were made with the South Asian communities through social networks and community-based non-religious organisations and the researcher (LM) attending informal talks at the community centers. There was no information to suggest how many people where approached and how many people did not want to take part. There was no information provided on any events, incidents activities, experiences or social processes which occurred during the course of the study.

<u>*Data Collection*</u><br>The data collection section was very brief, and did not have enough detail. It was stated that tape-recorded interviews were conducted. There was no discussion as to why interviews were chosen as the method of data collection. There was no information on how and under what conditions the research was collected. Issues related to rigour were addressed by assessing credibility – Participant validation was used to increase the probability that credible findings have been produced. Confirmability –An audit trail was maintained throughout the research process to enhance confirmability using a research diary and memos. Triangulation – Two levels of triangulation were used to improve validity and reliability in this study. Reflexivity – This acknowledges that researchers are part of the social world that is being studied.
<u>*Ethical Issues*</u><br>It was briefly stated that ethical and governance approval was secured from the appropriate organizations. No other ethical issues where discussed. The article was published in the International Journal of Nursing and health care of chronic illness Although ethical issues were not adequately discussed the authors will have confirmed ethical standards of the prior to publication.
<u>*Reflexivity of the Researcher*</u><br>Reflexivity was discussed due to the nature of the researcher's values, belief systems and interest may impact upon the research work that they are involved in. For this study, reflexivity was significant in relation to personal value systems as the author LM was a nurse, from a South Asian backgorund and with strong religious values and beliefs. Potential areas of role conflict including were identified such as practitioner–researcher conflict (the tendency to 'advise' people regarding effective management of diabetes and the prevention of long-term risks), feelings that cause the researcher to avoid or seek out situations (notably around language use of participants).
<u>*Data Analysis*</u><br>Data was analysed using the grounded theory. The guidelines for this approach were followed and data analysis was presented with sufficient detail and depth to provide insight into the experiences of the sample. Quotes were used to match themes derived from the data. To reduce bias two translators also analysed the transcripts as well as the researcher.
<u>*Findings*</u><br>The findings emerged from the experiences of the experiences of South Asian people with diabetes in the UK in relation to socio-cultural and dietary practices, religion and ageing influences on the perception and understanding of risks. They were discussed by providing example of the transcripts to back the themes that had been derived from the data. Member checking was employed as all the interviews were transcribed in English and the transcripts were returned to participants who could read English. The transcripts of the non-English-speaking participants were returned to the interpreters for validation. There was a clear description of the sample and the results enables us to appraise transferability. The researchers discussed how the study contributes to the understanding of the cohort under study. The findings of this study indicate how risk awareness resulted in either proactive or reactive risk engagement. Proactive risk engagement was unique to those with a strong internal LOC. Reactive risk engagement was predominantly influenced by sociocultural/religious beliefs and external LOC that operated in the affective domain among older South Asians with Type 2 diabetes.
<u>*Authenticity*</u><br>No stakeholders were involved in this research.
<u>*Fairness*</u><br>No stakeholders were involved in this research.
<u>*Promotion Of Justice*</u><br>No stakeholders were involved in this research.
**Jepson et al (2012)**

Qualitative Framework

The purpose and research question was to explore the barriers, motivators and facilitators to South Asian adults undertaking physical activity, with the broader aim of guiding the development of future interventions and services. The main aims of the research were to: describe the types of physical activities South Asians residing in the UK engage in; identify motivating and facilitating factors; to provide recommendations for the development of future services. The researchers employed sociology and anthropology theories of ethnicity, with sports sciences and public health perspectives. The importance and relevance of this study is the fact that there is currently little evidence of successful physical activity interventions amongst South Asian groups. It was not clear which philosophical framework the study took, however, a profile search revealed that the authors come from a background of nursing.

Study Setting

The study was conducted in a setting which was most convenient for the participants. Most of the focus groups took place in community centres though, one group met in a large centre café. The second author and the fourth author carried out the focus groups and interviews. It was not clear how long the focus group lasted. There were nine focus groups in total with 59 partcipants. This study employed sociology and anthropology theories of ethnicity.

This study did not require prolonged engagement or persistent observations in the setting to respond to the research questions. Enough information was provided to assess sufficient depth of data collection was carried out to permit an appreciation of the complexity of the phenomenon.

Study Design

Qualitative research design was used as the study aimed to gather a wide range of perspectives, views and experiences. The sample consisted of South Asian population as the researchers identified that there are is no evidence of successful interventions within this cohort. A variety of methods were used to recruit a representative sample. The main method of data collection was focus groups as the researchers stated that this method was appropriate to use for topic of physical activity as it was a not a sensitive subject and can be up for debate and discussion. Thematic analysis was used to analyse the results.

Sampling Procedures

Recruitment for the focus groups was through the urban centres of Aberdeen, Edinburgh and Glasgow. Purposeful sampling was used from groups who exercised together, mother and baby groups, prayer groups to achieve a sample that was representative to the South Asian population. Purposeful sampling was also used to recruit participants who were already involved in physical activities for example swimming to identify motivators and facilitators. It was stated that recruitment was thorough gatekeepers or existing contacts and for that reason it was not possible to identify who accepted or declined to participate in focus groups. It was identified that the study failed to recruit a Hindu group. Key informants who were recruited through public health lead in NHS Health Scotland were also interviewed to provide a range of viewpoints from policy through to community physical activity promotion paying particular attention to physical activity for British minority ethnic groups. There were 59 participants who took part in the focus groups and 10 key informants were interviewed. It was difficult to keep up with details on who accepted and who declined to take part as recruitment was through either contacts or 'gatekeepers'. All informants who where approached accepted to take part.

Data Collection<br>Data was collected through focus groups and supplemented with interviews with key informants. Interviews were digitally recorded and 9 focus groups were carried until the saturation of themes was reached which was in the final focus group. Triangulation took place as three researchers compared the themes and were also discussed with a wider team. There was sufficient detail on who was involved in each phase of the study as it was clear who collected the data, when the data was collected and who analysed the data. The researcher team consisted of individuals from a wide array of disciplines. The second author (FH) and the fifth author (GH) carried out the interviews and focus groups. The analysis was done by the first second and fifth author (RJ, FH & GA). They compared who compared and deliberated the themes to guarantee bias was minimised at the analysis stage. Themes were also discussed amongst the rest of the research team. Rigout was established members of the analysis team sharing and discussing transcripts and benefiting from a range of perspectives.
Ethical Issues<br>Ethical approval was granted by the Department of Applied Social Science (University of Stirling) Ethics Committee. There is an adequate consideration for all ethical issues, the article was published in the PLOS one Journal.
Reflexivity of the Researcher<br>The researchers have identified potential and actual biases. They used external people for sample recruitment and data was collected by women who did not have strong interest in physical activity. There was no indication as to the researcher's initial expectations of study results. Two members of the team were of South Asian origin. As the research team were from a variety of disciplines and backgrounds, this permitted to reflect on the data from a number of perspectives. However, reflexivity was considered as they were aware of possible bias and influences the research team can bring. Enough description of the researcher's roles was provided in the research design.
Data Analysis<br>The process of data analysis was presented with sufficient detail and depth to provide insight into the meanings and perceptions of the sample. Description of the data analysis was based on and was consistent with the method and purpose of the study. The researchers explained how the data was presented and selected from the original sample to demonstrate the analysis process. Sufficient data was presented to support findings. The research critically examined the researcher's individual roles, potential bias and influence during analysis and selection of data for presentation. Data was analysed using NVivo 7 package (QSR International's, 2006). Quotes were used to match concepts and themes derived from the raw data.
Findings<br>The findings emerge from the experiences of the sample and findings are explicit. There was an adequate discussion of the evidence both for and against the researcher's arguments. The researcher's discussed the credibility of their findings by discussing earlier studies and comparing it to them. Findings were discussed in relation to the original research question.<br>Member checking was not employed. Participants were not asked to confirm or disconfirm the accuracy of the researcher's observations and interpretations and were not asked their views about the credibility of the findings and interpretations.<br>The researcher's provide thick description of the sample and results by providing sufficient details about the context to assess whether the findings emerged from the data. They discussed that they tried to recruit a generalizable sample but can be difficult in qualitative research. They give examples of participants included in the study and use examples from the text to support conclusion. The researchers also discuss contributions the study makes to existing knowledge in relation to current policy, practice and research. This study identified several motivators and facilitators which influence part-taking in physical activity.
Authenticity<br>No stakeholders were involved in this research.

Fairness<br>No stakeholders were involved in this research.
Promotion Of Justice<br>No stakeholders were involved in this research.
**Gumbler (2014)**
Qualitative Framework<br>This study's aim and purpose was to investigate whether there was a knowledge gap among South Asian women with type 2 diabetes about diabetes development and management. The importance and relevance of this study was to understand of dietary and exercise recommendations was investigated, and other aims included assessing whether there were any language or cultural barriers present that affected the management of diabetes within this ethnic minority, with the hope of recommending possible culturally appropriate educational materials in bilingual languages to overcome any potential knowledge gap. This article stated why qualitative methods were appropriate as the research aims were most appropriately. This research seeks to interpret subjective experiences of research participants as they are looking individual experiences. It was not clear which philosophical framework the study took, however, a profile search revealed that the author come from a background of nursing.
Study Setting<br>The study setting has not been clearly described. Data was collected using semi-structured interviews at the participant's home, interviews lasted between 1.5 and 2 hours. The study used a qualitative research design but there was no indication as to what analysis was used.<br>This study did not require prolonged engagement or persistent observations in the setting to respond to the research questions. Enough information was provided to assess sufficient depth of data collection was carried out to permit an appreciation of the complexity of the phenomenon.
Study Design<br>The research design was not very clearly described. The researchers justified the research problem clearly but there was no justification as to what methods of analysis where chosen. The sample comprised Seven women had T2D age from 47 years to 73 years, aged from 31–76 years old. Immigrant status was related to participant age: first generation participants were 43–76 years (median 55), while second generation participants were 31–55 years (median 45). Data was collected through interviews. There was no clear indication of how data was analysed.
Sampling Procedures<br>The sampling selection was briefly described, patients were recruited if they were a South Asian female with type 2 diabetes. Recruitment was either in Warwickshire or in Birmingham through the local community by word-of-mouth over a 2-week period. There were no incentivise and potential participants were blinded to the aim of the study. Participants who volunteered were interviewed in English, Hindi, Gujarati or Punjabi depending on which language the interviewee preferred. There was no discussion on how many patients were approached, how many said no and how many chose not to take part. A topic guide was developed for the interviews, knowledge of the causes and prevention of diabetes. For example: What is diabetes? Why might an individual develop diabetes? How might one manage diabetes? Why do you think South Asians have an earlier onset of diabetes? What are the long-term effects of diabetes? The interview also included questions from the EQ-5D (EuroQol Group) questionnaire to investigate how diabetes impacted on their quality of life (mobility, self-care, usual activities, pain/discomfort, and anxiety/depression). The participants were also asked how diabetes affected other aspects of life (e.g. their sleep), and data of a typical day for the participants in terms of diet and physical activity were recorded. The interviewees were also asked about the care they received from healthcare professionals, whether they faced any difficulties in understanding instructions from healthcare professionals and whether they had any thoughts on how care for South Asian people could be improved. The participants' current height, weight, BMI, last dated HbA1c result, other medical conditions and family history of diabetes were recorded.

Data Collection

The data collection section was very brief, and did not have enough detail. It was stated that tape-recorded interviews were conducted. There was no discussion as to why interviews were chosen as the method of data collection. There was no information on how and under what conditions the research was collected. There was no discussion on triangulation or corroboration for data interpretation. It was not clear who was involved in each stage of the study. There was no reference to an audit trail for data collection, paper trail of field notes, interview transcripts, reflective/administrive journals or memos.

Ethical Issues

Although all ethical issues were not adequately discussed the authors will have confirmed ethical standards prior to publication, The article was published in Diabetes and Primary care Journal.

Reflexivity of the Researcher

The researcher did not identify potential and actual biases in the formulation of the research question, data collection, including sample recruitment and choice of location, initial expectations of study results, understanding of their roles within the production of knowledge. There no use of reflexive journals during and after data collection.

Data Analysis

The process of data analysis was not clearly presented there was no description and details on the process of data analysis. There were clear themes and matched quotes from the data. There was no contradictions taken into account and researchers role was not discussed.

Findings

The findings emerge from the experiences of the sample and findings are explicit. There was an adequate discussion of the evidence both for and against the researcher's arguments. The researcher's discussed the credibility of their findings by discussing earlier studies and comparing it to them. Findings were discussed in relation to the original research question. Member checking was not employed. Participants were not asked to confirm or disconfirm the accuracy of the researcher's observations and interpretations and were not asked their views about the credibility of the findings and interpretations.

The researcher's provide thick description of the sample and results by providing sufficient details about the context to assess whether the findings emerged from the data. The contribution this study makes is there is a considerable knowledge gap of the causes and effective management of type 2 diabetes among South Asians in the UK, particularly women.

Authenticity

No stakeholders were involved in this research.

Fairness

No stakeholders were involved in this research.

Promotion Of Justice

No stakeholders were involved in this research.

Rabiya Majeed-Ariss et al (2015)

Qualitative Framework

This study's aim was to explore the effects of type 2 diabetes on British-Pakistani women's identity and its relationship with self-management. The research question was stated clearly as this study wanted to explore 'How does having T2D affect British-Pakistani women's identity and how does this relate to their subsequent self-management?' For the purposes of this study, the concept of identity was understood according to Identity Theory (Burke & Tully 1977). The importance and relevance of this study was stated as the researchers identified that past research suggesting the need for the study. This article stated why qualitative methods were appropriate as the research aims were most appropriately. This research seeks to interpret subjective experiences of research participants as they are looking individual experiences. It was not clear which philosophical framework the study took, however, a profile search revealed that the author come from a background of psychology.

Study Setting<br>Qualitative research design was used as the study aimed to gather a wide range of perspectives, views and experiences from a sample of South Asian women. The study setting has not been clearly described. Interviews were conducted at the participant's home, there was no indication as to how long the interviews lasted.<br>This study did not require prolonged engagement or persistent observations in the setting to respond to the research questions. Enough information was provided to assess sufficient depth of data collection was carried out to permit an appreciation of the complexity of the phenomenon.
Study Design<br>The researchers justified the research problem clearly and described adequately. Data was collected from October 2009 to January 2010. Fifteen women had T2D and were British-Pakistani Muslims with children, living in Teesside, England. Participants' ages ranged from 31–76 years old. Immigrant status was related to participant age: first generation participants were 43–76 years (median 55), while second generation participants were 31–55 years (median 45). Data was collected using semi-structured interviews Thematic analysis was used to analyse the results (Braun & Clarke 2006).
Sampling Procedures<br>Participants were British-Pakistani adult women diagnosed with T2D living in Teesside, England. Purposive sampling (Maxwell 1997) was employed to provide diversity, recruitment was through English and Urdu language posters and participant information sheets advertising the study were displayed in mosques and Asian food shops. In addition, the researcher (RM) verbally introduced the study in Urdu at a local community There was no information to suggest how many people where approached and how many people did not want to take part. There was no information provided on any events, incidents activities, experiences or social processes which occurred during the course of the study.
Data Collection<br>The data collection section was very brief, and did not have enough detail. It was stated that tape-recorded interviews were conducted. There was no discussion as to why interviews were chosen as the method of data collection. There was no information on how and under what conditions the research was collected. There was no discussion on triangulation or corroboration for data interpretation. It was not clear who was involved in each stage of the study. There was no reference to an audit trail for data collection, paper trail of field notes, interview transcripts, reflective/administrive journals or memos. The interview schedule was informed by the findings of the systematic review (Majeed-Ariss et al. 2013) and the concept of identity.
Ethical Issues<br>It was stated approval for the study was secured from the University of Leeds, School of Healthcare Research Ethics Committee (SHREC/RP/165).Informed consent was obtained to undertake audio recorded interviews, no other ethical issues where discussed. The article was published in the International Journal of Clinical Nursing and the authors will have confirmed ethical standards prior to publication.
Reflexivity of the Researcher<br>The researcher did not identify potential and actual biases in the formulation of the research question, data collection, including sample recruitment and choice of location, initial expectations of study results, understanding of their roles within the production of knowledge. There no use of reflexive journals during and after data collection.

Data Analysis<br>Data was analysed using the thematic analysis. The guidelines for this approach were followed and data analysis was presented with sufficient detail and depth to provide insight into the experiences of the sample. Quotes were used to match themes derived from the data. The research team developed an initial coding frame informed by concepts including identity, confidence and roles. The coding frame was then applied flexibly to the remaining transcripts using QSR Nvivo to manage the data. Codes were constantly reviewed to ensure that they captured the meanings in the data. Similarities and differences within and across immigration and education status were considered.
Findings<br>The findings emerged from the exploration of the effects of type 2 diabetes on British-Pakistani women's identity and its relationship with self-management. They were discussed by providing example of the transcripts to back the themes that had been derived from the data. Member checking was not employed. There was a clear description of the sample and the results enables us to appraise transferability. The researchers discussed how the study contributes to the understanding of the cohort under study. The findings of this study The complex nature of BME patients' self-identification means this is an especially important consideration and one that may negatively influence self-management in this group. Such women may require continuing and intensive support to enable them to effectively live with and self- manage their diabetes.
Authenticity<br>No stakeholders were involved in this research.
Fairness<br>No stakeholders were involved in this research.
Promotion Of Justice<br>No stakeholders were involved in this research.
**Duthie-Nurse (1998)**
Qualitative Framework<br>This study's aim was discover the constraints that might make it difficult for particular groups to conform to modifications in diet.<br>The research question was not stated clearly and the importance and relevance of this study was stated as the author did not support the aim of the study with the current the literature. This article did not state why qualitative methods were appropriate and chosen, This research seeks to interpret subjective experiences of research participants as they are looking individual experiences.
Study Setting<br>Data was collected through interviews and were completed by the author. Three dietitians were key informants, however, their roles were unclear. Out of the 20 participants, 16 were interviewed at St Georges hospital, Tooting, 3 interviews took place in the participants home and 1 took place at a local café. There was no indication as to how long the interviews lasted. The philosophical framework the study took was through the anthropological framework. The author was from a nursing background.<br>This study did not require prolonged engagement or persistent observations in the setting to respond to the research questions. Enough information was provided to assess sufficient depth of data collection and to permit an appreciation of the complexity of the phenomenon.
Study Design<br>The research design was not very clearly described. The researchers justified the research problem clearly but there was no justification as to what methods of analysis where chosen. The sample comprised 20 Hindu women. Data was collected through interviews using a structured questionnaire with open and closed questions. There was no clear indication of how data was analysed.

Sampling Procedures<br>The sampling selection was briefly described, patients were recruited from St Georges Hospital, Tooting. The sampling strategy was not clearly described, though it was stated that 3 dietitians were approached as key informants and as the author had the largest caseload, those patients were chosen. There was no discussion on how many patients were approached, how many said no and how many chose not to take part. A topic guide was developed for the interviews, exploring views on diagnosis and diet. There was no information provided on any events, incidents activities, experiences or social processes which occurred during the course of the study.
Data Collection<br>The data collection section was very brief, and did not have enough detail. It was stated that interviews were conducted, however, it was unclear under what conditions the interviews took place. There was no discussion as to why interviews were chosen as the method of data collection. There was no information on how and under what conditions the research was collected. There was no discussion on triangulation or corroboration for data interpretation. It was not clear who was involved in each stage of the study. There was no reference to an audit trail for data collection, paper trail of field notes, interview transcripts, reflective/administrive journals or memos.
Ethical Issues<br>No ethical issues were discussed such as informed consent, privacy, and confidentiality and protection from harm. There was no discussion on how informed consent was obtained or a description of the procedure for confidentiality and privacy and how confidentiality and privacy was maintained during and after the study. Ethical adherence has been assumed due to the article being published in the Journal of Practical Diabetes International. The authors will have confirmed ethical standards prior to publication.
Reflexivity of the Researcher<br>There was no reflection of any potential bias in aspect of the study. It was unclear who was involved in each stage of the study. There was no indication if the researchers used a reflexive journal for data analysis, prior, during, and after data collection.
Data Analysis<br>The process of data analysis was not clearly presented there was no description and details on the process of data analysis. There were clear themes but there were no matched quotes from the data. There was no contradictions taken into account and researchers roles where not discussed.
Findings<br>Although there were no quotes, the results were from the experiences participants have faced with type 2 diabetes. There was no indication if member checking was employed. The contribution this study makes is the participants found it difficult to keep up with an appropriate diet, even though they had good knowledge of the complications associated with uncontrolled blood sugar levels. Most of the women felt a need to be seen as participating in the social and cultural values of people of their own age in their United Kingdom Asian community and, for them, these revolved largely around eating food.
Authenticity<br>No stakeholders were involved in this research.
Fairness<br>No stakeholders were involved in this research.
Promotion Of Justice<br>No stakeholders were involved in this research.
**Greenhalgh et al (1998)**

Qualitative Framework

The aim of this study was to explore the experience of diabetes in British Bangladeshis. A clearer goal was stated that the successful management of diabetes requires attention not just to observable behaviour but to the underlying attitudes and belief systems which drive that behaviour. The reason qualitative methods were used as the authors believed that the area understudy is relatively unexplored.

Study Setting

It was not clear where the study was conducted and length of time of the data collection. There was no indication if there was any trust building between the researchers and participants. However, there was a lengthy process of data collection as a range of methodological approaches were used in order to collect comprehensive data, such as audiotaped narrative, semi structured interviews, focus group discussions, construction of family tree, pile sorting exercise where disease ranking, foods, meal menus, "preferred" and "healthy" body size were ranked, structured vignette method, feedback of preliminary constructs to focus groups and study of patients general practice case notes.

Study Design

The researchers justified why they used the data collection methods as they wanted the participants to tell their story in their own words. The methods of analysis was not clearly justified they briefly mentioned that anthropological analysis was used as a framework. Anthropological analysis has three levels of cultural behaviour: (1) what people do (2) what they are actually observed to do (3) underlying belief system which drives that behaviour, however they do not justify why they used and the benefits it will have on the outcome of the study.

Sampling Procedures

Participants were recruited from three general practises in East London. Computerised diabetes registers and manual search of case notes were used to recruit participants apart from in one practise where opportunist sampling was used. However, they mentioned that out of the 44 subjects they approached, 40 of them agreed to take part but did not discuss why the four participants chose not to take part. There is no justification as to why they stopped recruiting. The sample consisted of 40 Bangladeshis, and a control group was recruited who lived in east London and had similar backgrounds, the control group consisted of eight white British and two afro-Caribbean participants.

Data Collection

The methods for data collection are consistent with the research question as they used a variety of methods to gain insight in the experiences of diabetes within the British Bangladeshis. Prompting questions were used for the interviews. However, there was no clear description under what conditions the research was collected. It was stated in the beginning of the article that interviews were tape recorded but there no clear description of how some of the other methods I.e. pile sorting exercise was conducted.

There was no explicit indication if there was a range of methods used for triangulation. It was mentioned that the interviews were translated and transcribed by an independent translator and were all checked for accuracy by the third author at the same time.

The third author collected the data, however there was insufficient detail on when and where the data was collected. All three authors had input on the analysis of the data. There was a brief mention that interviews were tape recorded, but there was no mention of an audit trail regarding, memos, note taking of decisions made in the study, reflective journals or administrative journals. Transcripts of interviews were mentioned as they used NUDIST software (N6, 2002) to analyse the transcripts.

Ethical Issues

The article was published in the British Medical Journal (BMJ) which states: *Statements regarding ethics approval; informed consent from participants*. (BMJ Publishing Group Ltd, 2014).There was a statement to indicate that a local ethics committee had approved the study and the authors will have confirmed to the required journal ethical standards prior publication. The authors will have confirmed ethical standards prior to publication.

Reflexivity of the Researcher<br>The researchers did not identify any potential and actual biases both as researcher and in the research design. The location they had chosen (East London) had a high proportion of Bangladeshi patients. The researchers did not indicate their initial expectations of study results, however they did use a topic guide for the semi-structured interviews. The roles of the researchers were the first author Trisha Greenhalgh supervised the study, wrote the paper, helped with the fieldwork, analysed and interpreted the paper. The Second author Ceceil Helman provided advice and contributed to the analysis and interpretation of the data and the third author A Mu'min Chowdhury performed the fieldwork, data entry, analysis and interpreted the data. The researchers did not indicate if any reflexive journal was used in data collection.
Data Analysis<br>There was a brief description on the analysis of the data, there no indication of the analysis process, and potential bias and influence during analysis and selection of data for presentation. Sufficient data was presented to support findings as quotes were used in the analysis section. The quotes match the concepts which align to the topic guide.
Findings<br>The findings are explicit and emerge from the experiences of the sample. There is an adequate discussion of the evidence both for and against the researcher's arguments. The researcher discussed the credibility of their findings and in relation to the original research question by taking into consideration and discussing the strengths and weaknesses of the study. The researchers indicated that the themes emerged from the information the participants provided from the experiences and that is how the constructs were developed.<br>There was no indication if member checking was employed as there was no description of participants being asked to confirm or disconfirm the accuracy of the researcher's observations and interpretations or ask their views about the credibility of the findings and interpretations.<br>The researchers provide thick description of the sample and results. The researchers discuss how the study contributes to existing knowledge about diabetes in this specific sample and it also discusses their findings in relation to current policy and practice. They also identify new areas where research is necessary such developing health promotion programmes which attempt to address practical barriers to positive health behaviours.
**Fleming, Carter & Pettigrew (2008)**
Qualitative Framework<br>This study's aim was to explore the influence of culture on diabetes self-management in Gujarati Muslim men. The importance and relevance of this study was stated as the researchers identified that past research suggesting the need for the study. This article stated why qualitative methods were appropriate as the research aims were most appropriately. This research seeks to interpret subjective experiences of research participants as they are looking individual experiences. It was not clear which philosophical framework the study took, however, a profile search revealed that the author come from a background of Nursing.
Study Setting<br>Qualitative research design was used as the study aimed to gather a wide range of perspectives, views and experiences from the sample. Observation was undertaken at the same time as the interview, and began then ended when the researcher was driving to and from the man's home. Following the interviews, the researcher was often invited to extend her visit and stay for refreshments. A further two separate sessions of observation were undertaken with one participant while he was attending a luncheon club and one participant participated in a nominal group (Delbecq et al. 1986) in the preliminary study. there was no indication as to how long the interviews lasted.<br>This study did not require prolonged engagement or persistent observations in the setting to respond to the research questions. Enough information was provided to assess sufficient depth of data collection was carried out to permit an appreciation of the complexity of the phenomenon.

Study Design

The researchers justified the research problem clearly and described adequately. Data were collected over a 11-month period, from June 2003–May 2004. A convenience sample of five men living with diabetes Men, rather than women, were chosen as the study group, because preliminary work had revealed that many older women could not speak fluent English. Funding and time constraints meant that EF, who could only speak English, had to undertake the fieldwork. Participants' ages ranged from 55–72 years old. Three of the men had migrated from Gujarat, India, and the other two from Uganda, East Africa. Data was collected using interview
and participant observation. Topic and analytic coding was used to analyse the results (Morse & Richards 2002).

Sampling Procedures

Participants lived in an urban area of northwest England, spoke English and identified themselves as Gujarati Muslim. A convenience sample of five men living with diabetes as recruited through attendance at luncheon clubs and contacts with community development workers.There was no information to suggest how many people where approached and how many people did not want to take part. There was no information provided on any events, incidents activities, experiences or social processes which occurred during the course of the study.

Data Collection

The data collection adopted a case-study approach. It was stated that interview
and participant observation were carried out. All, except for one of the men, who owing to ill health was able to participate in only one interview, participated in two interviews. The second interview enabled deeper insights into the participant's world to be gained and provided an opportunity to check the interpretation of the data collected previously. Observation was undertaken at the same time as the interview, and began then ended when the researcher was driving to and from the man's home. Following the interviews, the researcher was often invited to extend her visit and stay for refreshments. A further two separate sessions of observation were undertaken with one participant while he was attending a luncheon club and one participant participated in a nominal group (Delbecq et al. 1986) in the preliminary study. In total, there were 12 fieldwork sessions with these participants, which equated to 22 data pieces (nine interview transcripts, one nominal group transcript and 12 sets of observation notes). This research was undertaken as part of EF's doctoral studies. Therefore, she has taken the lead in the research process, while BC and JP provided supervision. The interview prompts were devised from a review of the literature and the findings of a preliminary study.

Ethical Issues

It was stated approval for the study was secured from the Faculty of Health and National Health Service ethics committees. Pseudonyms have been used, participant's personal details have been generalized and the area in which the research was undertaken has not been revealed. No other ethical issues where discussed. The article was published in the Journal of Nursing and Healthcare of Chronic Illness in association with Journal of Clinical Nursing and the authors will have confirmed ethical standards prior to publication.

Reflexivity of the Researcher

The researcher discussed potential and actual biases in the formulation of the research question, as a young white English woman, EF's identity (and to a lesser extent BC's and JP's identities) has been central in the interpretive process. As others have highlighted (Gardner 1995), there is always a risk that incorrect cross-cultural interpretations can be made, and in this study, we feel that the different researcher–participant cultures, mother tongue language and ethnicity may have heightened this risk. By undertaking a member check, collecting data which are detailed and rich and undertaking several cycles of reflective analysis, this risk has been minimized.

Data Analysis<br>Data was analysed using topic and analytic coding were used in the analysis process. The guidelines for this approach were followed and data analysis was presented with sufficient detail and depth to provide insight into the experiences of the sample. Quotes were used to match themes derived from the data. This type of coding was undertaken before analytical coding, with the intention of it enabling immersion in the data. Analytic coding is a natural progression from topic coding (Morse & Richards 2002), as it involves looking for deeper meanings in the data. Hence, the analytic codes were more abstract, whereas topic codes were often more descriptive. Three cycles of analysis were undertaken, during which, the researcher moved between the processes of data coding, literature reviewing, theory building and fieldwork. Hence, the analysis process was a cyclical one, which required 'flexibility' and 'creativity' (Carter 2004, p. 87).
Findings<br>The findings emerged from exploring the influence of culture on (type 2) diabetes self-management in Gujarati Muslim men. They were discussed by providing example of the transcripts to back the themes that had been derived from the data. It was stated that member checking was employed, however, it was not reported what methods were used. There was a clear description of the sample and the results enables us to appraise transferability. The researchers discussed how the study contributes to the understanding of the cohort under study. The findings of this study highlighted the dissonance between the approach taken in some government policy and research, and an embodied and dynamic approach to providing culturally appropriate care. The reductionist perspective is theoretically and empirically questionable, and we call for the inclusion of a dynamic embodied approach. Such an approach could be used as a basis for developing and delivering culturally appropriate research, practice and policy.
Authenticity<br>No stakeholders were involved in this research.
Fairness<br>No stakeholders were involved in this research.
Promotion Of Justice<br>No stakeholders were involved in this research.
**Patel & Iliffe (2016)**
Qualitative Framework<br>This study's aim was to To explore the influence of health beliefs and behaviours on diabetes management in British Indians, as successful management of diabetes is dependent on<br>underlying cultural beliefs and behaviours. The importance and relevance of this study was stated as the researchers identified that past research suggesting the need for the study. This article stated why qualitative methods were appropriate as the research aims were most appropriately. This research seeks to interpret subjective experiences of research participants as they are looking individual experiences. It was not clear which philosophical framework the study took, however, a profile search revealed that the author was a Medical Student.
Study Setting<br>Qualitative research design was used as the study aimed to gather a wide range of perspectives, views and experiences from the sample. The study setting has not been clearly described. It was not indicate where the interviews were conducted, Interviews lasted between 25 and 50 min<br>This study did not require prolonged engagement or persistent observations in the setting to respond to the research questions. Enough information was provided to assess sufficient depth of data collection was carried out to permit an appreciation of the complexity of the phenomenon.

Study Design<br>The researchers justified the research problem clearly and described adequately. Data were collected between March and April 2015. British Indians with type 2 diabetes registered with a single practice in North London were invited by their general practitioner to take part in interviews about the management of their diabetes with a medical student working in the practice (V.P.). Participants' average age was 61. There were a total of 10 participants and the<br>with eight males and two females. Data was collected using a mixed methodology using semi-structured interviews and a pile-sorting exercise. A thematic analysis approach was undertaken.
Sampling Procedures<br>British Indians with type 2 diabetes registered with a single practice in North London were invited by their general practitioner to take part in interviews about the management of their diabetes with a medical student working in the practice (V.P.).They were provided with an information sheet about the study and invited to express interest. All those who responded positively were interviewed, after written consent had been obtained.There was no information to suggest how many people where approached and how many people did not want to take part. There was no information provided on any events, incidents activities, experiences or social processes which occurred during the course of the study.
Data Collection<br>It was stated that within the interview, a structured vignette was used to assess participants' knowledge of diabetes. The vignettes were only used to inform the interviews and no<br>attempt was made to categorise participants according to their knowledge. Face-to-face interviewing was used as it allowed the use of different data collection techniques. There was no discussion on triangulation or corroboration for data interpretation. This research was undertaken by the first author were the second author provided supervision. There was no reference to an audit trail for data collection, paper trail of field notes, interview transcripts, reflective/administrive journals or memos. An interview schedule was written with open-ended questions about different aspects of diabetes management.
Ethical Issues<br>It was stated ethical approval for this research was granted by the NHS Research Ethics Committee in February 2015 (REC Reference: 15/NW/0181). Permission was sought from the NHS Trust Research & Department (R&D) and NHS R&D assurance was granted in March 2015 (R&D Reference: 171109). Written consent had been obtained. No other ethical issues where discussed. The article was published in the Primary Health Care Research & Development and the authors will have confirmed ethical standards prior to publication.
Reflexivity of the Researcher<br>The researcher did not identify potential and actual biases in the formulation of the research question, data collection, including sample recruitment and choice of location, initial expectations of study results, understanding of their roles within the production of knowledge. There no use of reflexive journals during and after data collection.
Data Analysis<br>A thematic analysis approach was used. There no indication of the analysis process, and potential bias and influence during analysis and selection of data for presentation. Sufficient data was presented to support findings as quotes were used in the analysis section. The quotes match the themes.

Findings<br>The findings emerged from exploring explore the influence of health beliefs and behaviours on diabetes management in British Indians, as successful management of diabetes is dependent on underlying cultural beliefs and behaviours. They were discussed by providing example of the transcripts to back the themes that had been derived from the data. It was not stated if member checking was employed. There was a clear description of the sample and the results enables us to appraise transferability. The researchers discussed how the study contributes to the understanding of the cohort under study. The findings of this study highlighted that health beliefs and behaviours are not static but always changing. Different populations have different beliefs and behaviours which play an integral role in the management of diseases. An understanding of health beliefs should be used as a start point for culturally sensitive diabetes education. Furthermore, it should be noted that this is not only an issue that needs to be tackled by primary health care but also by public health as<br>well given the importance of social networks and the desire for more sharing of experiences.
Authenticity<br>No stakeholders were involved in this research.
Promotion Of Justice<br>No stakeholders were involved in this research.
**Prinjha, Ricci-Cabello et al. (2020)**
Qualitative Framework<br>This study's aim was to to explore the perceptions and views of British South Asian patients with T2D on mHealth SMS text messaging to support medication adherence. The importance and relevance of this study was stated as the researchers identified that past research suggesting the need for the study. This article stated why qualitative methods were appropriate as the research aims were most appropriately. This research seeks to interpret subjective experiences of research participants as they are looking individual experiences. It was not clear which philosophical framework the study took, however, a profile search revealed that the author was from an Anthropology background.
Study Setting<br>Qualitative research design was used as the study aimed to gather a wide range of perspectives, views and experiences from the sample. The study setting was clearly described. Eight exploratory focus groups were conducted around Leicester, The focus groups lasted between 1.5-2hours. This study did not require prolonged engagement or persistent observations in the setting to respond to the research questions. Enough information was provided to assess sufficient depth of data collection was carried out to permit an appreciation of the complexity of the phenomenon.
Study Design<br>The researchers justified the research problem clearly and described adequately. Data were collected between September 2017 and March 2018. British South Asian populations include first, second, and third generation people of Indian, Pakistani, Bangladeshi, and Sri Lankan descent. Participants were purposively sampled to include a broad range of views. Participants' age range was between 18-84. There were a total of 67 participants. Data was collected using foucs groups. A thematic analysis approach was undertaken.
Sampling Procedures<br>Potential participants were informed about the focus groups verbally and with written information in English by a CBMEH project support worker and by community center managers. There was no information to suggest how many people where approached and how many people did not want to take part. There was no information provided on any events, incidents activities, experiences or social processes which, occurred during the course of the study.

<u>*Data Collection*</u><br>Data was collected through focus groups. There was sufficient detail on who was involved in each phase of the study as it was clear who collected the data, when the data was collected and who analysed the data. The analysis was done by the first (SP) Transcripts were then independently coded by NN. All four research team members (SP, RC, NN, and AF) discussed the data and themes, and finalized themes using a consensus process. Discrepancies were resolved and agreed by consensus. There was no reference to an audit trail for data collection, paper trail of field notes, transcripts, reflective/administrive journals or memos.
<u>*Ethical Issues*</u><br>It was stated ethical approval for this research Ethical approval was obtained from the University of Oxford Central University Research Ethics Committee (Ref R50751/RE001). No other ethical issues where discussed. The article was published in JMIR Mhealth Uhealth and the authors will have confirmed ethical standards prior to publication.
<u>*Reflexivity of the Researcher*</u><br>The researcher did not identify potential and actual biases in the formulation of the research question, data collection, including sample recruitment and choice of location, initial expectations of study results, understanding of their roles within the production of knowledge. There no use of reflexive journals during and after data collection.
<u>*Data Analysis*</u><br>A thematic analysis approach was used. There no indication of the analysis process, and potential bias and influence during analysis and selection of data for presentation. Sufficient data was presented to support findings as quotes were used in the analysis section. The quotes match the themes.
<u>*Findings*</u>
The findings suggests the varied needs of British South Asian patients in relation to short messages to support DSM. A mHealth SMS designed for the general population, can be acceptable and relevant to UK South Asian populations but may need to include additional content with culturally adapted messages about South Asian foods, natural and herbal approaches used in the United Kingdom and South Asia, safe medicine taking when fasting, and exercise in women-only groups—messages that can be available to those who opt to receive them from a system that can provide individual choice.
<u>*Authenticity*</u><br>No stakeholders were involved in this research.
<u>*Fairness*</u><br>No stakeholders were involved in this research.
<u>*Promotion Of Justice*</u><br>No stakeholders were involved in this research.
**Pardhan et al. (2018)**
<u>*Qualitative Framework*</u><br>The aim of this research was to determine whether barriers to diabetes awareness and self-help differ in South Asian participants of different demographic characteristics (age, gender, and literacy) with type 2 diabetes living in the United Kingdom. The importance and relevance of this study was stated as the researchers identified that past research suggesting the need for the study. This article stated why qualitative methods were appropriate as the research aims were most appropriately. This research seeks to interpret subjective experiences of research participants as they are looking individual experiences. It was not clear which philosophical framework the study took, however, a profile search revealed that the author was from a medical background.

Study Setting<br>Qualitative research design was used as the study aimed to gather a wide range of perspectives, views and experiences from the sample. The study setting was clearly described. It was not clear how long the focus groups lasted. This study did not require prolonged engagement or persistent observations in the setting to respond to the research questions. Enough information was provided to assess sufficient depth of data collection was carried out to permit an appreciation of the complexity of the phenomenon.
Study Design<br>The researchers justified the research problem clearly and described adequately. It was not clear when data was collected. Five focus group discussions were held in community centers in Peterborough where the groups and one focus group discussion for literate older females living in Cambridge was held in their research facilities at Anglia Ruskin University, Cambridge campus. Data was collected using focus group discussions. A thematic analysis approach was undertaken.
Sampling Procedures
South Asian communities from local contacts, leaflets, advertisements, faith leaders (e.g. Muslim Sheikhs, Hindu priests), and social gatherings were approached. Participant information sheets were sent to potential participants in advance of the focus group discussions. The participant information sheet, consent forms and adverts were translated in native language (Urdu, Hindi, Nepali and Gujarati) for people who were illiterate. There was no information to suggest how many people where approached and how many people did not want to take part. There was no information provided on any events, incidents activities, experiences or social processes which occurred during the course of the study.
Data Collection<br>Data was collected through focus groups. There was sufficient detail on who was involved in each phase of the study as it was clear who collected the data, when the data was collected and who analysed the data. Discrepancies were resolved and agreed by consensus. There was no reference to an audit trail for data collection, paper trail of field notes, transcripts, reflective/administrive journals or memos.
Ethical Issues<br>It was stated ethical approval for this research was granted Ethical approval was obtained from the Faculty (of Medical Science) Research Ethics Panel, Anglia Ruskin University prior to data collection. No other ethical issues where discussed. The article was published in Ethnicity and Health and the authors will have confirmed ethical standards prior to publication.
Reflexivity of the Researcher
The researcher did not identify potential and actual biases in the formulation of the research question, data collection, including sample recruitment and choice of location, initial expectations of study results, understanding of their roles within the production of knowledge. There no use of reflexive journals during and after data collection.
Data Analysis<br>A thematic analysis approach was used. There was no indication of the process, and potential bias and influence during analysis and selection of data for presentation. Sufficient data was presented to support findings as quotes were used in the analysis section. The quotes match the themes.
Findings<br>The findings suggest that various demographics (age, gender, literacy) of participants showed different barriers to improved diabetic awareness, self-help and health care access in South Asian patients with type 2 diabetes. Most participants, irrespective of the demographic characteristics, reported lack of awareness and availability of ethnically/culturally tailored educational diabetic

programs. Temptation to give into the wrong kind of food, especially during weddings/festivals, was reported more often by females than in males. Males were more dependent on females to supply the right kind of food. More use of community centres was highlighted, mostly by the illiterate older participants, for improving awareness about diabetes and increasing physical activity.
Authenticity<br>No stakeholders were involved in this research.
Fairness<br>No stakeholders were involved in this research.
Promotion Of Justice<br>No stakeholders were involved in this research.

Table S6. Supporting Evidence for categories: Example quotation extracts from articles

**Synthesising Argument : Decisional Conflict for Self-management Behaviours**
**Synthetic construct: Diet & food**<br><br>a) *Understanding choices*<br>• All six participants associated bananas, potatoes and apples with a worsening of blood glucose levels. This perception led them to consume fewer fruits and vegetables than the recommended "5 a day". [69]<br>• ['I am fine the way I am. I have a bit of eye problem and my (blood) pressure but nothing else.mmh…I am doing things…I go to the clinic and the lady in the clinic tells me all this' (participant 12)]. Another felt that information on diabetes was not very useful in general ['even when I control my diet I feel the levels are high and sometimes when I am bad my levels are good. I can't understand it… he (diabetes) goes up and down when he (diabetes) feels like it' (participant 7)]. [61]<br>• In most families tea is drunk along with a small portion of puffed rice or biscuits as breakfast. Generally tea is prepared by boiling tea leaves, milk, sugar and water in a sauce pan and the strong and thick brew is served in cups. [63]<br>b) *Cultural identity*<br>• Immigrant Bangladeshis have retained to a very high degree their traditional ethnic dietary habits. Any changes made by the British Bangladeshis in this study appeared to be in the form of elaboration of the traditional customs rather than adaptation to the host culture. [63]<br>• They don't like to eat our foods all the times. They try to eat this country's food all the time. But I insist them to eat our foods otherwise they wouldn't grow healthy. Moreover they need to learn our culture. [71]<br>• 'The thing is us Asian people we mostly eat roti, which is the truth, but we should not eat that much roti, it is not good for the sugar. But the thing is roti is the foundation food for us, we are used to it now It we were to eat fast food all day and not eat roti then we'd not be able to sleep'. (Mrs Qureshi, Pakistani Muslim, 60s) …Mr Mohan, an Indian Hindu in his 60s, described how 'we need cooked foods and spices, because we've been brought up on that'. 58]<br>c) *Cooking methods*<br>• In my opinion some kind of a diet plan should be introduced showing, for example, if you eat this amount of food in the morning, and in the afternoon one small bowl of lentils and one chapatti for example... If there was a diet plan, we could look at it and follow it. That would help....But these diet plans and portion sizes don't exist for Punjabi diets. They don't exist for our diets and foods. [76]<br>• most also described being reluctant to change the cooking practices with which they were familiar, and which were used on the Indian subcontinent, such as frying vegetables in oil before making them into curries. As Mr Idress put matters simply, 'I don't think our food was made to be boiled y'know', a sentiment echoed by Mr Maskeen who explained that, 'you know our kinds of foods, fried food is part of that and a lot of foods are fried'. [58]<br>• Different modes of cooking are seen to modify edibility in different ways, altering either strength (for example through dilution by water, or through addition of `strong' constituents such as ghee and spices) or digestibility (through the softening of texture through boiling/steaming or the compression of substance through baking). Cooking methods are varied to suit the different perceived needs of the partakers and the social function of the meal. The dominant mode of cooking is for everyday meals is boiling and shallow frying. In cooking the emphasis is on taste and flavour rather than on visual presentation. Because of this there is a marked tendency to make liberal use of oil, ghee, and spices, especially when entertaining or celebrating. Their generous use is seen as a mark of afluence and hospitality. [63]

**Synthetic construct: Physical Activity**<br><br>*a) Social-cultural norm*<br>• many families appeared to do little physical activity together, partly owing to the work commitments of the men. Working in shops or as taxi drivers, for example, meant that they rarely had time off at weekends to spend with their wives and children. Several men also commented on the significant amount of time that they spent at their religious centre (mosque or temple); those that did take exercise as a family talked about activities such as badminton and walking. [70]<br>• Many female respondents had been brought up in India and Pakistan, and only migrated to Britain as adults (normally for the purposes of marriage and/ or to join husbands who had already moved to Britain). These women found it particularly difficult to increase their physical activity as they had not been socialized into spending time outdoors, let alone to participating in sports, when they were younger…'I don't go out, nor do I have the habit of going out. I just read my namaz [prayer] and sit in my own home'. [57]<br>• While some women favoured dancing to Bollywood videos or taking part in Bhangra dancing, one woman raised religious concerns: F3: ''I don't like doing that [Bhangra dancing] to music because it is not allowed in Islam, Indian music and songs. I don't want me or my family to participate in that, you know.' [70]<br>*b) Endorsement*<br>• One key informant also felt that religious centres could take on a leadership role in promoting physical activity: ''And I sometimes think even religious groups in terms of the work I do, if religious institutions could encourage it and in partnership with what they teach, because active… being fit and something is not discouraged at all, no religion says it's bad, it's encouraged because essentially it gives you a better and healthier life.'' [70]<br>• Others stuck to a day-to-day exercise regime at home and took up hobbies which increased their levels of activity: 'I exercise every day, I wake up every day at 4.15am and do 1 hour treadmill every day. Every day, Monday to Friday. Then Saturday I do gardening, I love my garden. It is a very good hobby for me and it is a good form of exercise too'. [74]
**Synthetic construct: Medication**<br><br>*a) Management*<br>• Respondents expressed some initial trepidation about taking OHAs, as they perceived this change to their regimen as signifying that their condition had deteriorated and that they had taken on the identity of a sick person: "I was devastated [about being prescribed glipizide]. I wasn't happy at all. But it was explained to me that diabetes always progresses, no matter how careful you are." (R1, Pakistani, female) "If you start taking them, you become a patient." [56]<br>• 'Rushing off in the morning, for example to get ready for my grandchildren to prepare them for school, keeps me very busy and I get tired, I then forget to take medicine' [75]<br>*b) Alternative*<br>• A number of participants reported controlling their diabetes by eating bitter foods. ['I know that eating ginger is good and garlic is good. It thins my blood… if you eat bitter things then it 'thingies' (balances) the sugar in your body' [61]<br>• Although the majority of patients were aware of the anti-diabetic properties of the bitter foods and herbs, few used them specifically as therapeutic agents. [57]<br>• A majority of the participants used certain vegetables to control their diabetes. The most common were karella (also known as 'Momordica charanita' or bitter melon) and fenugreek:'I was watching this program on the TV by a Yoga Swami [Hindu Priest] and he was saying the bitterness of karella is good for diabetics'. [74]

**Synthesising Argument: Management strategies and Factors influencing Conflict**
**Synthetic construct: Social Conflict**<br><br>*a) Family support & role*<br>• These women talked about how South Asian cultural expectations could make them feel isolated or misunderstood:-Some days I want to meet people but often I don't go out because of the diabetes. I worry what the wider family would say. . . "she was ill so why did she go out?" I think that, so I don't bother going out too often. Rabiya Majeed-Ariss et al (2015)<br>• Acts of commensality (i.e., shared food consumption) were also described as extending to the wider community. Respondents described a culture of gift-exchange in which luxury food items, such as Pakistani and Indian sweets, were given/received on occasions such as the completion of exams or the passing of a driving test, as well as at special events, such as a marriage. [63]<br>• She went on to describe how financial hardships and family responsibilities impinged on treatment choices and asserted that taking exercise was difficult for her because of the arthritis she experienced. [59]<br>*b) Motivational support*<br>• and then went on to elaborate saying that his daughters '… ask me to come to their houses, you know … especially on Saturday or Sunday.' He also explained that '… the youngest daughter, the oldest daughter, they also understand about everything [regarding his diabetic diet]' and so they prepare him a separate dish to the other family members by '… taking all sweet out and … in my dish would be the sugar-free.' [67]<br>• There were instances, however, of both men and women with a strong internal LOC, taking responsibility for their diabetes and being convinced that there was something that they could do to manage it. In these instances, the common thread that ran across in most cases was that they were strongly motivated individuals and there was either a medical professional within the family or that the individuals themselves were educated and fluent in English: My uncle was a doctor. My father was a doctor in India and I had Hygiene and Physiology. So, I think I knew the general basics myself. (Padma, from India – Son and daughter in law are GPs and live very close to where she lives) [65]
**Synthetic construct: Religious obligations**<br><br>• Fasting during Ramadan, going on 'Hajj' and attending prayers regularly were given a very high priority, especially among men. There were instances of people fasting at whatever cost which involved taking insulin and adjusting their meal timings accordingly as their religion requires them to do so and there were other instances where women especially chose not to fast on health grounds as there were other forms of dispensation that could be made instead, as described in the following quote. I pray and do other things you know, but fasting, I don't do fasting but I know that because of my illness and our religion allows it if you are ill, you don't have to fast but lots of people I know in the community, they have diabetes but they are still fasting. [64]<br>• Most respondents, however, claimed to have devised the strategy of cutting down themselves (as Mr Sheikh put it, 'I am the doctor'), in some cases, in light of their experiences of fasting during religious events such as Ramadan (the ninth month of the Muslim calendar, in which Muslims fast between dawn and sunset) and Karva chot (a Hindu event requiring a wife to fast for a whole day to grant her husband a longer life). On such occasions, respondents noted improvements in their blood glucose control, leading them to deduce that cutting down their food intake could be a quick and effective means of controlling their diabetes when their blood glucose levels were high. Mr Awan, for instance, observed that his blood glucose had fallen to 'normal' levels following Ramadan. This not only promoted him to explain to his 'astounded' doctor that 'I'd been fasting this year, like I did last year', but also to suggest to the interviewer that 'I will try to continue the way I've been eating during Ramadan'. [58]

- The influence participants felt they had to manage diabetes and the decisions made in handling risks were also centred around their personal faith and spiritual values. Diabetes was viewed as a trial from God. These trials were not only believed to be from God but also as a source of reward in their 'life after death'. Their belief that their life on earth was temporal and there was an eternal reward when they were affected by diseases, empowered them with the courage to pursue carrying on with their diabetes as part of their temporal lives: So, I think depending on my faith, predestination these are tests and trials from God. So, He tests you in one way or the other. So, this is one way, He's testing me perhaps. There is a reward you know, reserved for you for the life after death. So, actually, more and more trials and tribulations that you go through, more reward is there…[65]

Synthetic construct: Healthcare delivery/ relationship

- My GP doesn't discuss it [diabetes] at all. when I go to the nurse I am not given that much information' 'I would like more (information)' 'I want more (information) because it's better to have more (information)' 'All she (the nurse) says is do more exercise and then they increase the medications and decrease it'[75]
- Participants reported that the NHS could improve services by providing more information in bilingual formats or using simpler language (i.e. less medical terminology) on English information leaflets, and training staff on giving culturally appropriate advice to South Asian people with diabetes. [69]
- Some, like Mrs Saeed, a Pakistani Muslim in her 30s, claimed that they had received very limited information: 'they don't tell you how to eat, they just say ''do diet''', others, especially veteran patients, complained that the advice proffered had been insensitive to their culture and food preferences. For instance, Mrs Akbar, a Pakistani Muslim in her 60s, described how, when she had been diagnosed 10 years previously, 'they [dietitians] would be like ''stay away'' if you even mentioned chapatti and curries'. [58]

Synthetic construct: Health Beliefs, language & literacy

a) *T2D knowledge*

- Only one participant (Participant 2) knew the medical reasons for the onset of diabetes (i.e. either not enough insulin is produced by the body or the insulin produced is not effective). Participant 3 described diabetes as: "a bad thing preventing individuals from enjoying themselves." Participant 1 described diabetes as: "a disease in which you cannot consume sugar and potatoes."[69]
- A particularly striking feature of the interviews is that when respondents talked about why they thought that they had got diabetes (and other diseases) they almost universally attributed the causes to factors outside their control. [57]
- Others attributed their diabetes to a spiritual fate, as in the following quote. Actually we don't believe in these things. You know, how in this country they do believe like I should have been in good diet from childhood then I might not be getting it. We don't believe in that. Our fate is totally different. It's like you think, Allah decided to give you the illness, He will. No matter how well you are or how healthy you are. [64]

b) *T2D timescale & social evaluation*

- There were several situations described by the participants which required a decision to be made based on weighing up different risks. This often happened in relation to eating practices, where the risk of causing social offence had to be considered alongside the risk of less than ideal diabetes management. In both of the quotes below, the participant describes how the diabetes affects the individual's ability to socialize. When she goes to somebody's house you know, she cannot say that 'I'm hungry. I need to eat', because she is on Insulin and she has to see to the time of her meals. Same thing with the parties, she has to avoid the parties because of this. (Woman with diabetes, F6)

Any social ceremony or if you go to visit somebody, you will carry one or two kilos of sweets with you, you know. If you take healthy food like fruit, etc., they will look down on you that: 'They have insulted us and brought fruits to our house when they came'. It's that sort of perception. [64]

- Extended families on the other hand, were perceived as less supportive by some participants of both generations. These women talked about how South Asian cultural expectations could make them feel isolated or misunderstood: -Some days I want to meet people but often I don't go out because of the diabetes. I worry what the wider family would say. . . "she was ill so why did she go out?" I think that, so I don't bother going out too often. Khalida – 1st generation -Last time somebody passed away I had to go to London and the food they gave us I wasn't happy with it. I thought, well that's no good for me. . . the flour they'd used was white and you know, the curry they gave us was like loaded with a pile of "ghee" on it and I'm looking at it and thinking "I can't eat this". And then I can't remember who was next to me and she goes, "shut up and just have it". [73]
- Indeed, other respondents also highlighted the seemingly obligatory nature of acts of commensality within their families and communities. In these, declining to eat roti, metai (traditional sweets) and other foodstuffs could not only result in their being 'the odd man out' as Mr Rahul an Indian Hindu in his 60s put it, but, more crucially, could cause 'offence' as Mrs Saeed described. Indeed, Mrs Parween, a Pakistani Muslim in her 60s, told the interviewer how she always accepted and ate metai when it was offered by neighbours and kin because: 'They [would] wonder why you don't want it when they have offered it to you. Maybe they think it's because you don't like them. That's why I take it'.[57]

c) *Communication*

- We find it difficult to understand English. Information (is) not provided in local language [75]
- Some, like Mrs Saeed, a Pakistani Muslim in her 30s, claimed that they had received very limited information: 'they don't tell you how to eat, they just say ''do diet''', others, especially veteran patients, complained that the advice proffered had been insensitive to their culture and food preferences. For instance, Mrs Akbar, a Pakistani Muslim in her 60s, described how, when she had been diagnosed 10 years previously, 'they [dietitians] would be like ''stay away'' if you even mentioned chapatti and curries'.[58]
- the doctor tells me and others as well… umm… to eat vegetables and to eat fruit. But I don't like eating it all the time' (participant 6); 'I know that eating red meat will make your diabetes (levels) go high'. [61]
